# Two Different Virulence-Related Regulatory Pathways in *Borrelia burgdorferi* Are Directly Affected by Osmotic Fluxes in the Blood Meal of Feeding *Ixodes* Ticks

**DOI:** 10.1371/journal.ppat.1005791

**Published:** 2016-08-15

**Authors:** Sébastien Bontemps-Gallo, Kevin Lawrence, Frank C. Gherardini

**Affiliations:** Laboratory of Zoonotic Pathogens, Rocky Mountain Laboratories, National Institute of Allergy and Infectious Diseases, National Institutes of Health, Hamilton, Montana, United States of America; University of Montana, UNITED STATES

## Abstract

Lyme disease, caused by *Borrelia burgdorferi*, is a vector-borne illness that requires the bacteria to adapt to distinctly different environments in its tick vector and various mammalian hosts. Effective colonization (acquisition phase) of a tick requires the bacteria to adapt to tick midgut physiology. Successful transmission (transmission phase) to a mammal requires the bacteria to sense and respond to the midgut environmental cues and up-regulate key virulence factors before transmission to a new host. Data presented here suggest that one environmental signal that appears to affect both phases of the infective cycle is osmolarity. While constant in the blood, interstitial fluid and tissue of a mammalian host (300 mOsm), osmolarity fluctuates in the midgut of feeding *Ixodes scapularis*. Measured osmolarity of the blood meal isolated from the midgut of a feeding tick fluctuates from an initial osmolarity of 600 mOsm to blood-like osmolarity of 300 mOsm. After feeding, the midgut osmolarity rebounded to 600 mOsm. Remarkably, these changes affect the two independent regulatory networks that promote acquisition (Hk1-Rrp1) and transmission (Rrp2-RpoN-RpoS) of *B*. *burgdorferi*. Increased osmolarity affected morphology and motility of wild-type strains, and lysed Hk1 and Rrp1 mutant strains. At low osmolarity, *Borrelia* cells express increased levels of RpoN-RpoS-dependent virulence factors (OspC, DbpA) required for the mammalian infection. Our results strongly suggest that osmolarity is an important part of the recognized signals that allow the bacteria to adjust gene expression during the acquisition and transmission phases of the infective cycle of *B*. *burgdorferi*.

## Author Summary

*Borrelia burgdorferi*, the Lyme disease agent, exploits a multifaceted enzootic cycle that requires a tick vector for successful transmission between mammalian hosts. Two different regulatory systems control genes that are required to complete this infective cycle. The Hk1/Rrp1 two-component system affects genes required for successful transfer between mammal and tick vector while the Rrp2-RpoN-RpoS regulatory cascade modulates genes essential for the transmission from the tick to a new vertebrate host. Data presented in this study indicate that fluctuations in osmolarity in the tick midgut directly affect these two regulatory pathways. Osmolarity in the lumen of the tick adjusts to the osmolarity of the incoming blood (blood meal) to promote water and ion flux into tick tissues. A positive water flux is essential to generate sufficient saliva for prolonged feeding. We propose that *B*. *burgdorferi* uses this physiological parameter as an important signal to adapt and regulate genes required for survival in the tick (through Hk1/Rrp1) and transmission to a new host (through Rrp2-RpoN-RpoS).

## Introduction

*Borrelia burgdorferi*, the Lyme disease agent, survives and grows in mammals and various vertebrate hosts. However, the bacteria are not transmitted directly to a new host. Instead they are acquired by a hematophagous arthropod (*Ixodes scapularis)* and transmitted to a new host. Cycling between a host and the vector requires the bacteria to adapt and survive in both mileus. The environment of the host is well defined: nutrient-rich, constant temperature, stable pH, established ion concentrations and osmolarity [1]. Overall, the mammalian host provides the bacteria with a very steady environment for survival provided that they can successfully evade an aggressive host immune system. In contrast, the tick presents a more variable environment with parameters that are gradually changing before, during and after feeding. Acquisition of *B*. *burgdorferi* begins when uninfected ticks begin feeding on infected mammals. Initially, this colonization is characterized by rapid growth of the bacteria and regulation of gene expression by the two-component system (TCS), Response Regulator 1 (Rrp1) and Histidine Kinase 1 (Hk1) [2–4]. As the blood meal is consumed, a feast-famine succession that lasts for several weeks slowly converts *B*. *burgdorferi* from rapid growth to stationary phase. During this progression, *Borrelia* adjusts gene expression for long-term survival via regulatory networks mediated by Rel_Bbu_ (RelA/SpoT homolog), the *Borrelia* oxidative stress regulator (BosR) and σ^S^ (RpoS) [5–7]. After molting to the next developmental stage, the ticks begin the next feeding and parameters in the midgut revert. *B*. *burgdorferi*, localized specifically to the midgut, begin to grow and alter gene expression according to reconstituted feeding conditions (replenished nutrients, temperature, etc.). Some of these conditions act as signals to upregulate key virulence and transmission factors (OspC, DbpA, BBA66, etc.) via the Rrp2/RpoN/RpoS regulatory cascade [8, 9].

The way the midgut environmental conditions affect the expression of this regulatory system and virulence factors required for the successful transmission has been extensively studied [10]. In several cases, *in vitro* conditions have been used to mimic parameters that are suspected to exist or have been measured in the tick midgut or the blood meal [11–15]. In addition, the transcription of virulence related genes has been assayed directly from *B*. *burgdorferi* RNA extracted from feeding ticks [16]. Interestingly, one parameter that has been completely overlooked is osmolarity. Because of the extended feeding time of *Ixodes* ticks (5 to 6 days), water from the blood meal must be recycled through the hemolymph to the salivary glands to generate adequate saliva for prolonged feeding. This water flux is followed by a corresponding flux of ions such as Na^+^, K^+^ and Ca^2+^. In *Dermacentor andersonii*, Kaufman and Phillips demonstrated, by directly measuring the ion concentration, that the osmolarity changes throughout feeding [17–19]. Early studies suggest that, in *Ixodes ricinus*, the salivary glands function in osmoregulation and facilitate the recycling of 70% of the water from the blood meal to the salivary glands [20, 21]. These studies suggest that *B*. *burgdorferi* should encounter osmotic conditions in the feeding tick midgut that are generated by water and ion flux necessary to produce the saliva required for successful feeding.

Bacteria respond to the physiological changes associated with changes in osmolarity by a process known as osmoadaptation [22–24]. Osmoadaptation is classically associated with the synthesis or uptake of a limited set of molecules called compatible solutes [22, 25, 26]. There are two categories of compatible solutes: solutes that have no effect on growth, and those that do have an effect on growth (osmoprotective molecules) [27]. Bacteria use osmoprotective molecules to modulate their intracellular osmolarity so they can grow and divide [22, 28]. In *E*. *coli*, the increasing K^+^ concentration is directly related to the increase of the environmental osmolarity [29, 30]. Potassium is also known to activate key regulatory proteins that are involved in the regulation of intracellular pH [31, 32]. Bacteria also accumulate, by transport or *de novo* synthesis, specific amino acids like proline or glutamate [15, 22, 27, 33]. The major role of the glutamate is to offset the uptake of K^+^, which inhibits enzyme activity [32, 34].

In this study, we determined the effects of changes in osmolarity on the virulence and physiology of *B*. *burgdorferi*. First, we measured the osmolarity (mOsm) in the bloodmeal, saliva and hemolymph isolated from feeding ticks, then tested *B*. *burgdorferi* cells for their ability to grow over a range of osmolarities. Surprisingly, *Borrelia* was only able to grow normally between 250 mOsm and 650 mOsm, which very closely matched the range of osmolarity in the bloodmeal during tick feeding (~300–600 mOsm). The growth, morphology and motility were dramatically affected by osmolarity outside of this narrow range. Interestingly, at low osmolarity, *Borrelia* cells expressed increased levels of virulence factors (OspC, DbpA) required for successful transmission. Finally, we analyzed the osmoadaptation by following the expression of the genes putatively involved in osmoregulation (*proU*, *gltP*, etc.), or virulence (*ospC*, *dbpA*, etc.) at various osmolarities. Mutants that did not express the putative L-glutamate transporter (*gltP*) or proline transport (*proU*) system were more sensitive to changes in osmolarity than wild-type cells, suggesting that L-glutamate and proline were osmoprotective molecules. We hypothesize that bloodmeal osmolarity may directly affect the expression of key virulence factors and may serve as a physiological signal to trigger *B*. *burgdorferi* to migrate from the midgut to the salivary glands during transmission.

## Results

### The midgut osmolarity in adults and nymphs fluctuated during feeding

**Fig 1:**
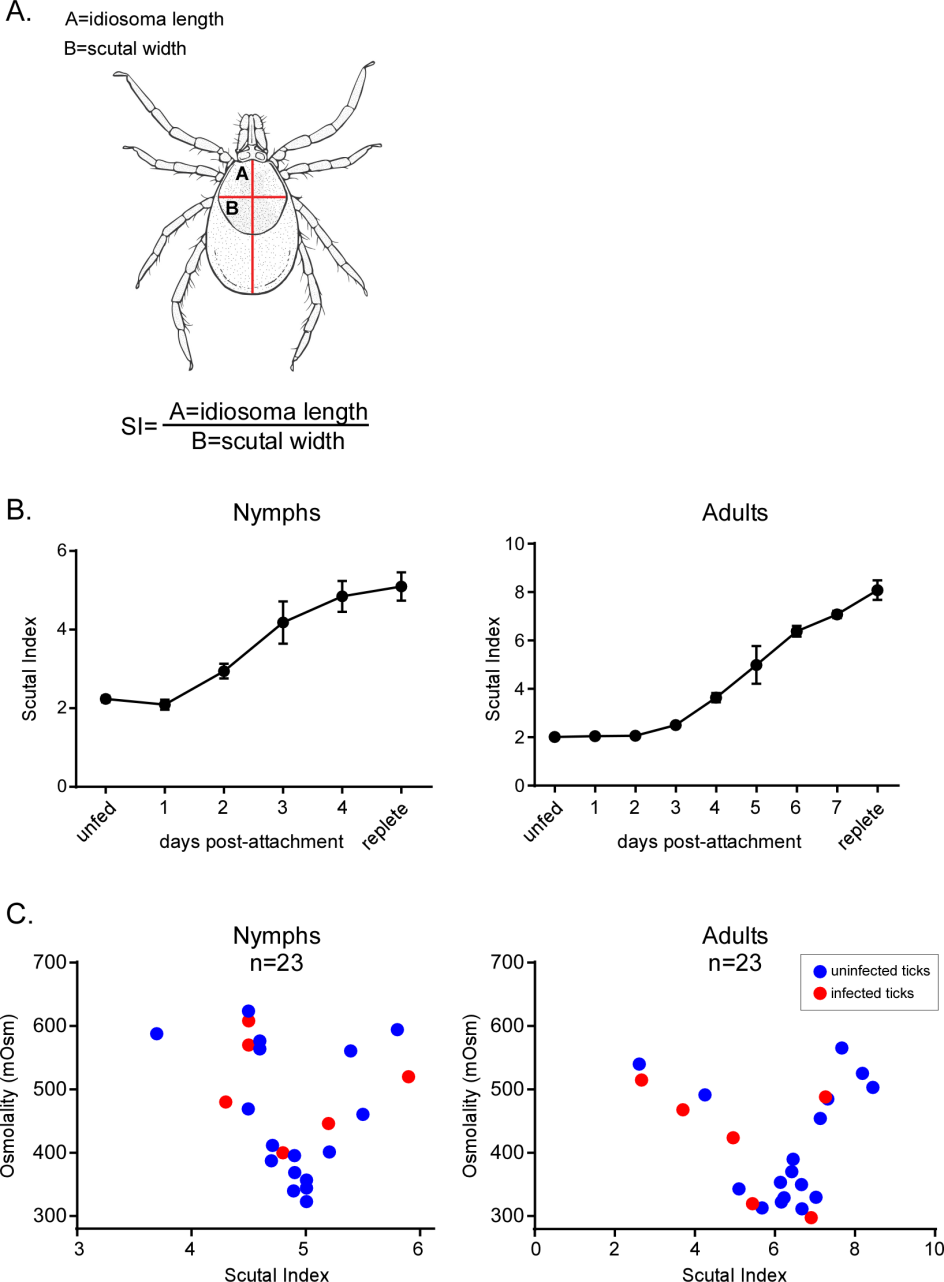
*B*. *burgdorferi* tolerates a narrow range of osmolarity (250 to 650 mOsm). (A) A drawing of an *I*. *scapularis* tick showing the measurements used to determine scutal index: A = idiosoma length, B = scutal width [35]. (B) Development of the scutal index during feeding of nymph and adult ticks. Each point represents the average of the measurements of 15 ticks. (C) Osmolarity of the blood meal isolated from the midgut of nymph or adult ticks during the feeding as a function of the scutal index. Each red circle denotes the osmolarity measured in the blood meal of an infected, feeding tick. Each blue circle denotes the osmolarity measured in the blood meal of an uninfected, feeding tick. Osmolarity was measured with Wescor vapor pressure osmometer as described in the Methods section.

Based on previous observations in *I*. *ricinus* and *D*. *andersonii* [17–20], we hypothesized that the osmolarity of the *Ixodes* tick midgut changes throughout its feeding cycle. To test this hypothesis, we measured the osmolarity of the midgut contents, hemolymph and saliva of *I*. *scapularis* in feeding nymph or adult ticks (Fig 1). Initially, we harvested feeding ticks from host animals at specific times (days) after attachment to analyze midgut contents. However, because of a lack of consistency in feeding efficiency between individuals in a feeding cohort, we decided to use scutal index to measure feeding progress. The scutal index is the ratio of the length of the idiosoma to the maximum width of the scutum (see methods, Fig 1A and 1B) [35] and this proved to be an effective method to evaluate the duration of feeding. In both nymph and adult ticks, it was not possible to measure the osmolarity of midgut contents before 24 h of the initiation of feeding due to the extremely low volume of recoverable midgut material.

Therefore, feeding was interrupted by detaching ticks, scutal index were measured, the midgut contents were extracted, osmolarity was measured and the data were plotted as a function of the scutal index (Fig 1C) [35]. In adult ticks, the osmolarity began at ~ 550 mOsm at a scutal index of 2 and then decreased to ~300 mOsm (with the lowest value measured at 264 mOsm) at a scutal index between 5–7. Finally, the osmolarity increased as feeding finished, and in replete ticks, returned to an osmolarity of ~550 mOsm at a scutal index between 7–8 (Fig 1C, Adults). We also measured the changes in osmolarity in the midguts of feeding nymphs. Again, we were unable to measure the osmolarity at early time points. As the scutal index reached 3.5–4, the osmolarity approached ~600 mOsm and then decreased to 300 mOsm at a scutal index of 5 (Fig 1C, Nymphs). Again, as observed in the adult ticks, the osmolarity rebounded to ~500–600 mOsm at the conclusion of feeding. These data showed a very similar pattern of amplitude and fluctuation in osmolarity in the midgut of feeding adults and nymphs.

We also measured the osmolarity of mouse (335 mOsm ± 2.8) and rabbit (304.7 mOsm ± 9.5) blood confirming previously published values [1]. Additionally, hemolymph and saliva were collected from feeding ticks and the osmolarity of biological triplicate samples were measured. Hemolymph (311.4 mOsm ± 33.3) and saliva (323.5 mOsm ± 6.4) had osmolarities very similar to those measured in host blood. Taken together, these data suggest that *B*. *burgdorferi* encounters little change in osmolarity in the hemolymph, saliva or in the mammalian host but faces a variation in osmolarity (~275–600 mOsm) in the tick midgut during feeding.

### *B*. *burgdorferi* will only grow within a narrow range of osmolarity

**Fig 2:**
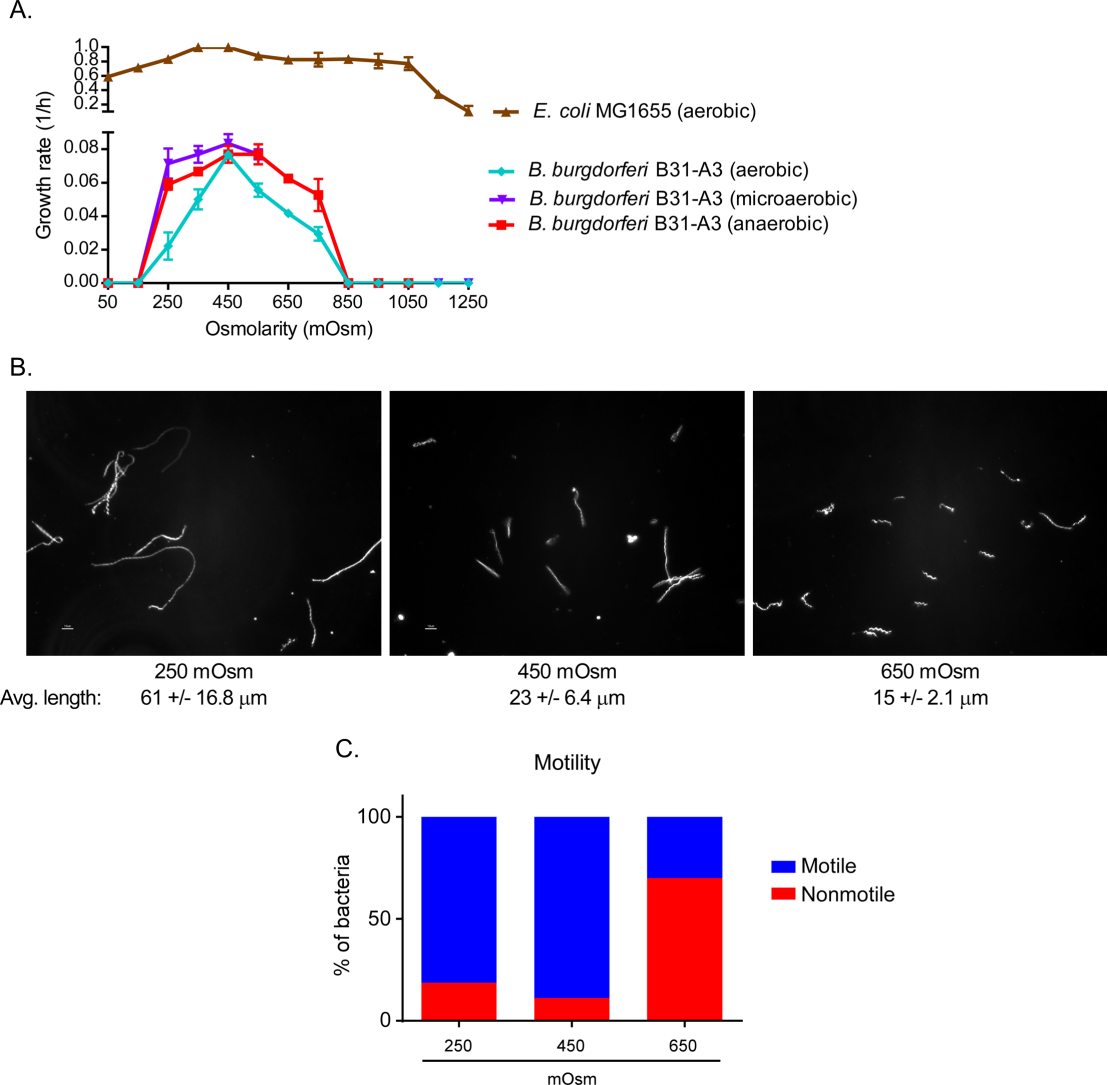
The effects of osmolarity on the physiology, cell morphology and motility of *B*. *burgdorferi*. (A) Rate of growth of the wild-type strain B31-A3 from 50 to 1,250 mOsm under anaerobic (90% N_2_, 5% CO_2_, 5% H_2_), microaerobic (90% N_2_, 5% CO_2_, 5%O_2_) and aerobic (78% N_2_, 21% O_2_, 0.05% CO_2_) conditions at 34°C and *E*. *coli* in aerobic conditions at 34°C. (B) Spirochetes, grown at different osmolarities, were examined by dark-field microscopy. Under each photograph of cells is the average length of 200 cells from 5 independent cultures (C) The motility of cells was evaluated by assessing 200 cells per culture condition (e.g., 250 mOsm) by dark-field microscopy.

After characterizing the osmolarity in the tick and mammalian blood, we attempted to understand if this dynamic affected *B*. *burgdorferi* growth and physiology. Considering that most bacteria and some spirochetes (*Leptospira*) tolerate a wide range of osmolarity [22, 24, 36], we were skeptical that the range of osmolarity observed in the tick midgut would have much effect on *B*. *burgdorferi*. To define the range of osmotolerance of *B*. *burgdorferi*, we monitored the growth rate in BSK-II medium at various osmolarities (150 to 1,250 mOsm) and in different concentrations of oxygen (Fig 2A, S1 Fig). As a control, we also monitored the growth rate of *E*. *coli* MG1655 in LOS medium over the same osmolarity range. Unlike *E*. *coli*, which can tolerate a range of osmolarity between 50 to 1,050 mOsm, *B*. *burgdorferi* was only able to grow between 250 and 650 mOsm in microaerobic or anaerobic conditions, with an optimal growth rate between 250 and 550 mOsm. At osmolalities <200 mOsm and >750 mOsm, most of the cells lysed. Transferring “survivors” to fresh BSK-II media (450 mOsm) indicated that these cells could not recover. Under aerobic conditions, *B*. *burgdorferi* was more sensitive to the osmolarity (Fig 2A). These data indicated that *B*. *burgdorferi* could tolerate a relatively narrow range of osmolarity (250 and 650 mOsm). However, considering the range of osmolarities in the feeding tick, it seems that *B*. *burgdorferi* is well adapted to survive in the tick midgut environment. Because of these results, all subsequent experiments were done between 250 and 650 mOsm under microaerobic conditions.

### Changes in osmolarity directly affected motility and cell morphology

During evaluation of the growth rates of *B*. *burgdorferi* at different osmolarities, we observed an effect on motility in higher osmolarity. In addition, cell morphology also was affected. High and low osmolarity are known to have a global effect on the cell physiology and gene regulation in many bacteria [24]. In *B*. *burgdorferi*, cellular morphology is critical for proper motility as the cells utilize endoflagella to perform waveform motility [37]. To understand how differing osmolarities affect these aspects of *B*. *burgdorferi* physiology, we observed the morphology and motility of cells at the three physiologically relevant osmolarities: 250, 450 and 650 mOsm using dark-field microscopy at mid-log phase of growth (4–5 X 10^7^ cells/ml) (Fig 2B and 2C). At 450 mOsm, the cells displayed normal morphology, *i*.*e*. long waveform-shaped cells (Fig 2B). At lower osmolarity (250 mOsm), the cells were slightly longer, with normal motility (Fig 2C). At an osmolarity of 650 mOsm (Fig 2B), ~80% of the cells were non-motile and ~10% had altered motility (twitching) (Fig 2C), and were shorter (Fig 2B). We confirmed by plating that non-motile cell were viable. These observations suggested that osmolarity affected both cellular morphology and motility in *B*. *burgdorferi*. The changes in cell shape could indicate an adaptation to a change in water flux. The observed effects on motility at higher osmolarity may reflect physical constraints on flagellar function or may indicate an effect on membrane potential and/or cellular energy.

### Decreasing osmolarity triggers changes in *B*. *burgdorferi* protein profile

**Fig 3:**
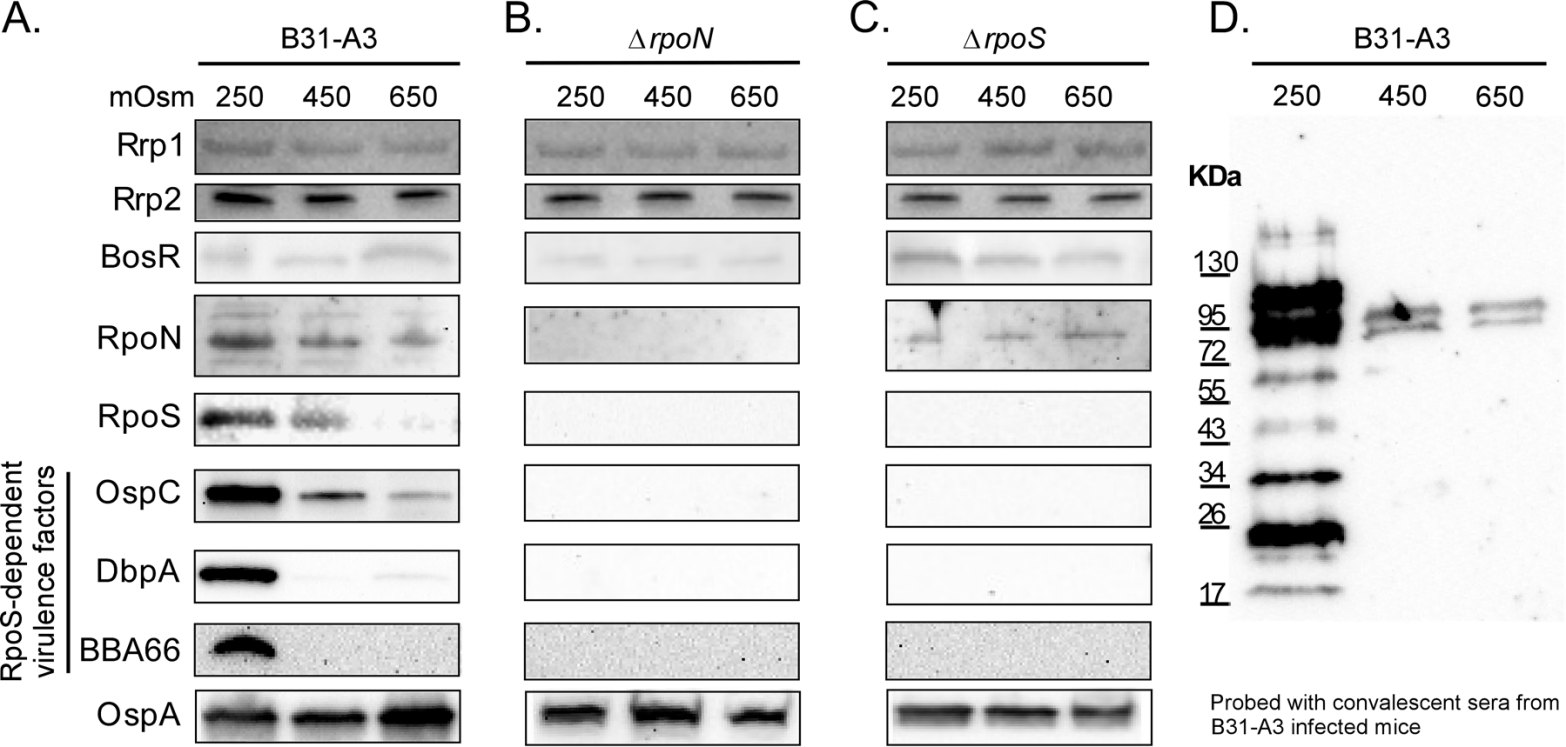
The effects of osmolarity on specific proteins regulated by the RpoN-RpoS regulatory cascade. *B*. *burgdorferi* strains B31-A3, B31-A3Δ*rpoN* and B31-A3Δ*rpoS* were grown in 250, 450 and 650 mOsm BSK-II to mid-log phase and cell lysates (40 μg of protein/lane) were analyzed by immunoblotting. (A) B31-A3 lysates (40 μg of protein/lane) were probed with OspA, OspC, DbpA, BBA66, RpoN, RpoS, BosR, Rrp1 and Rrp2 antigen-specific antisera. (B) B31-A3Δ*rpoN*, and (C) B31-A3Δ*rpoS* cell lysates probed with the same antigen-specific antisera. (D) B31-A3 (40 μg protein/lane) cell lysates probed with serum from mice infected by *B*. *burgdorferi* via tick bite.

Because of the observed changes in osmolarity in the feeding tick midgut, we analyzed the production of key proteins involved in successful transmission at different osmolarities. The levels of virulence factors OspC, DbpA and BBA66 increased at an osmolarity of 250 mOsm while OspA increased slightly at higher osmolarity (650 mOsm) (Fig 3A, S2 Fig). Key regulatory proteins involved in the regulation of these virulence related proteins were also assayed. The levels of Rrp2, Rrp1 and BosR did not change significantly at any osmolarity tested. However, RpoN and RpoS, which have been shown to regulate these and other virulence factors, increased at an osmolarity of 250 mOsm (Fig 3A, S2 Fig). More importantly, we directly tested the production of virulence factors in strains B31-A3Δ*rpoN* and B31-A3Δ*rpoS* at different osmolarities (Fig 3B and 3C, S2 Fig). While these mutants grew normally at all osmolarities tested compared to wild-type B31-A3, no changes were observed in the production of OspC, DbpA or BBA66 when the mutants were grown at 250 mOsm. These data strongly suggest that the RpoN-RpoS regulatory cascade was involved in the regulation of these virulence factors at lower osmolarity. Also, immunoblots of cell lysates of *B*. *burgdorferi* grown at 250, 450 and 650 mOsm were probed using serum from mice infected with *B*. *burgdorferi* B31-A3 by tick bite (Fig 3D). Spirochetes grown at 250 mOsm, corresponding to the osmolarity measured at the midpoint in a feeding tick bloodmeal, in tick saliva or in mammalian blood, showed increased reactivity with infected serum (Fig 3D).

**Fig 4:**
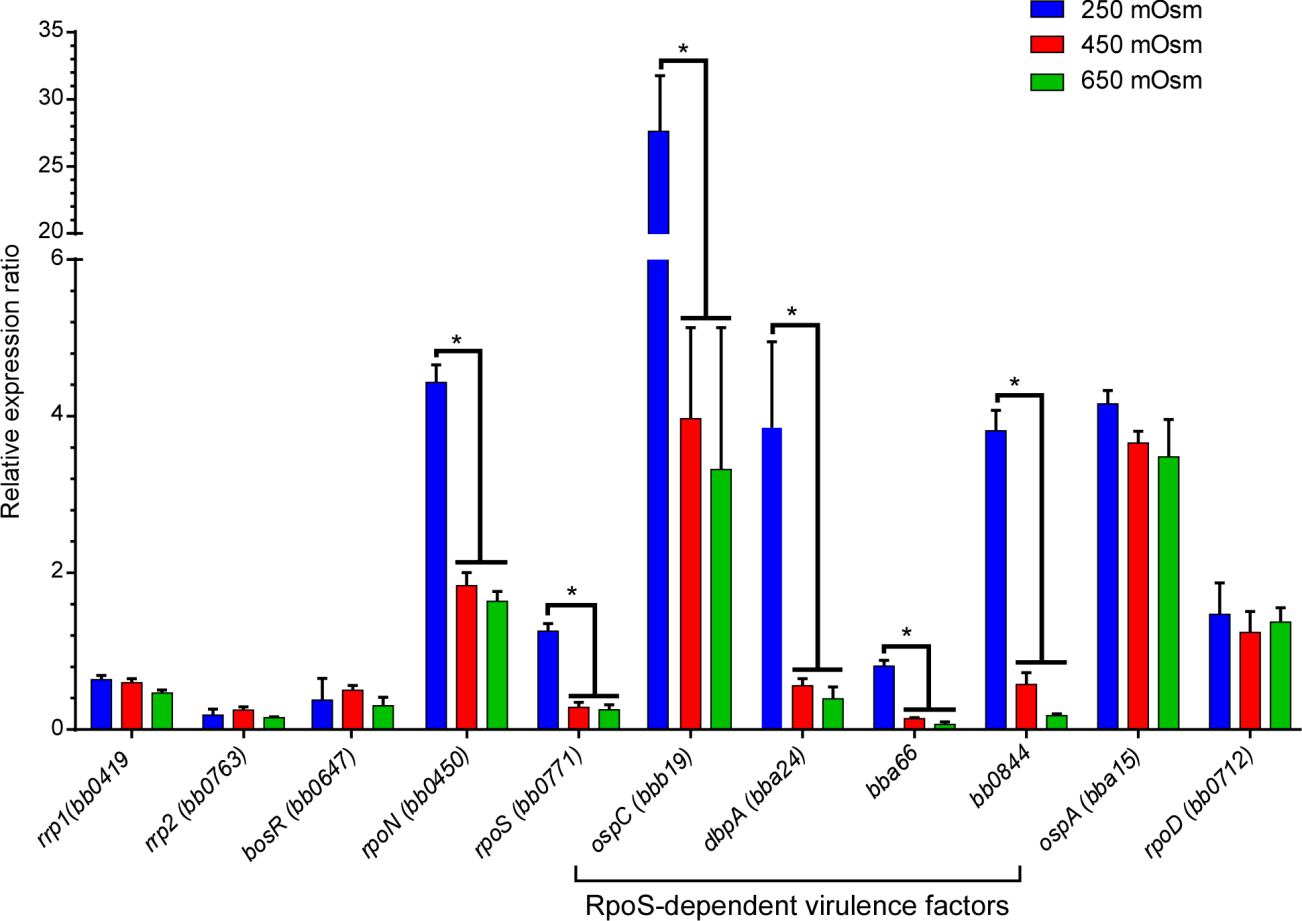
Gene expression of genes encoding crucial regulatory proteins and virulence factors at 250, 450 and 650 mOsm. The expression of genes encoding regulatory proteins (*rrp1*, *bosR*, *rrp2*, *rpoS*, *rpoN*), virulence factors (*ospC*, *dbpA*, *bba66*, *bb0844*, *ospA*) and *rpoD* analyzed by qRT-PCR in *B*. *burgdorferi* B31-A3. RNA isolated from cells grown at 250, 450 and 650 mOsm. The gene expression was normalized to *enoS*.

We also tested the expression of the genes encoding these proteins by qRT-PCR. At 250 mOsm, similar to osmolarity that was measured in the blood, at the mid-point of feeding and in tick saliva, expression of the sigma factors *rpoN* and *rpoS* increased 2.5 and 4.5-fold respectively (Fig 4). We measured the expression of RpoS-dependent virulence factors (*ospC*, *dbpA*, *bba66*, *bb0844*) and found that similar to the *rpoN*-*rpoS* expression pattern, the expression of these four genes increased significantly (6.9, 5.9, 5.7 and 6.8-fold, respectively) at 250 mOsm (Fig 4). Although both *rpoN* and *rpoS* showed changes in gene expression in response to lower osmolarity, the sigma factor, *rpoD*, did not change in response to osmolarity (Fig 4). Regulation of *rpoS* and RpoS is transcriptional, translational and post-translational [7, 8, 38–41]. Expression analysis of the Borrelia oxidative stress regulator (BosR), which is thought to directly regulate *rpoS*, indicated that there was no change in transcription or translation of *bosR* in response to changes in osmolarity (Figs 3 and 4). Additionally, the transcription and translation of *rrp2*, which is required to activate the RpoN-RpoS cascade, was not affected by osmolarity. Since both BosR and Rrp2 are transcriptional activators, their regulatory effects on RpoN and RpoS might only require activation of these proteins (e.g., oxidation of BosR, phosphorylation of Rrp2) rather than an increase in the transcription or translation of the genes encoding them. Taken together, these data indicate that lower osmolarity could trigger an increase in the expression of key virulence factors in actively growing cells and this increase was directly linked to the RpoN-RpoS regulatory cascade.

### Rrp1 was required for the survival of *B*. *burgdorferi* at higher osmolarity

*B*. *burgdorferi* cells colonizing ticks are exposed to a distinct range of osmolarities during the tick lifecycle (Fig 1C). Previous studies show that the Hk1-Rrp1 TCS is required for tick midgut colonization. *hk1* or *rrp1* mutants are unable to be acquired by ticks fed on infected mice or introduced by artificial feeding [2]. Because TCSs are known to be involved in osmoregulation (e.g., OmpR) [42], we investigated the possibility that Rrp1 might be involved in the adaptation of *B*. *burgdorferi* to tick midgut osmolarities.

**Fig 5:**
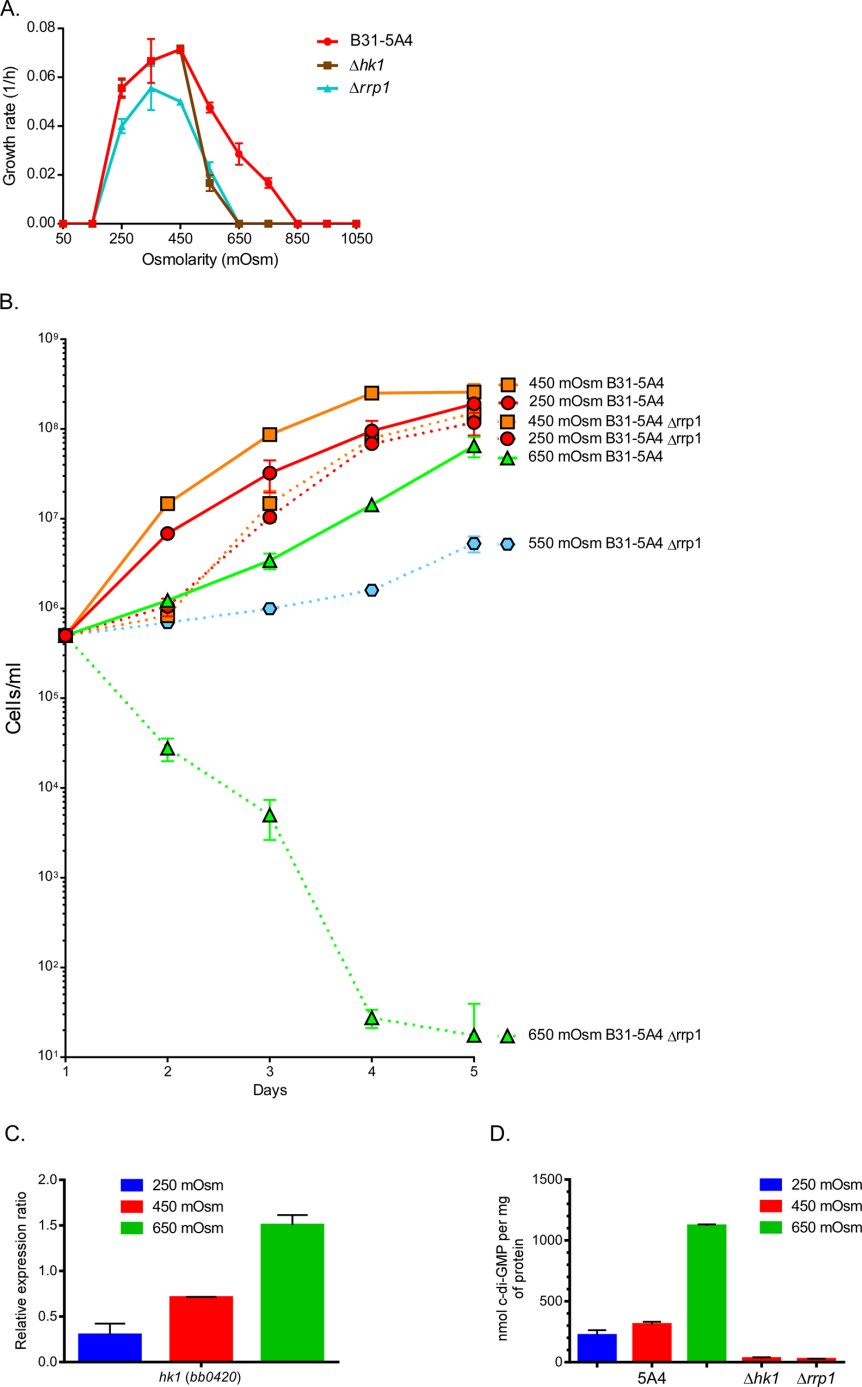
Rrp1 is required for the survival of *B*. *burgdorferi* at higher osmolarity. (A) Rate of growth of B31-5A4, B31-5A4Δ*hk1* and B31-5A4Δ*rrp1* mutants at osmolarities ranging from 150 to 1050 mOsm under microaerobic growth conditions. (B) Growth of strain B31-5A4 and 5A4Δ*rrp1* mutant in BSK-II at various osmolarities. Cells were quantified by colonies on BSK-II plating media. (C) *hk1* expression was analyzed by relative qRT-PCR in *B*. *burgdorferi* B31-A3 normalized to *enoS*. (D) Quantitation of c-di-GMP in B31-5A4, B31-5A4Δ*hk1* and B31-5A4Δ*rrp1* mutants.

To test our hypothesis, we monitored the growth rate of *B*. *burgdorferi* strains 5A4, 5A4 Δ*hk1* and 5A4 Δ*rrp1* at 250, 450, 650 mOsm (Fig 5A and 5B). The mutant strains were not affected at low osmolarity (250 mOsm) but were dramatically affected at osmolarities >550 mOsm. In fact, Δ*rrp1* mutant cells in BSK-II media at increased osmolarity lysed completely mimicking the phenotype that has been reported for these mutant strains in ticks (Fig 5B)[2]. Further, *hk1* expression has been shown to increase in the bacteria during acquisition by the tick from the host [2]. In this study, *hk1* expression increased 5-fold as osmolarity increased from 250 to 650 mOsm (Fig 5C). In contrast, *rrp1* and Rrp1 expression did not change at the osmolarities tested (Figs 3 and 4). However, because Rrp1 has been shown to have diguanylate cyclase activity, we measured the levels of cyclic di-GMP (c-di-GMP) in cells at different osmolarities. At 650 mOsm, the intracellular levels of c-di-GMP increased from 220 nM/mg protein (250 mOsm) to 1120 nM/mg protein (Fig 5D). These data suggest that: i) Hk1 and Rrp1 are required for the transition of *B*. *burgdorferi* from the mammal (300 mOsm) to the initial conditions in the tick midgut at the beginning of feeding (600 mOsm), ii) Rrp1 enzymatic activity dramatically increases at 650 mOsm, iii) Hk1 and Rrp1 could be sensing changes in osmolarity, and iv) c-di-GMP could act as an effective secondary messenger for the successful acquisition and long-term survival of *B*. *burgdorferi* in ticks.

### The *proU* locus responded to changes in osmolarity and was constitutively expressed throughout the enzootic cycle

**Fig 6:**
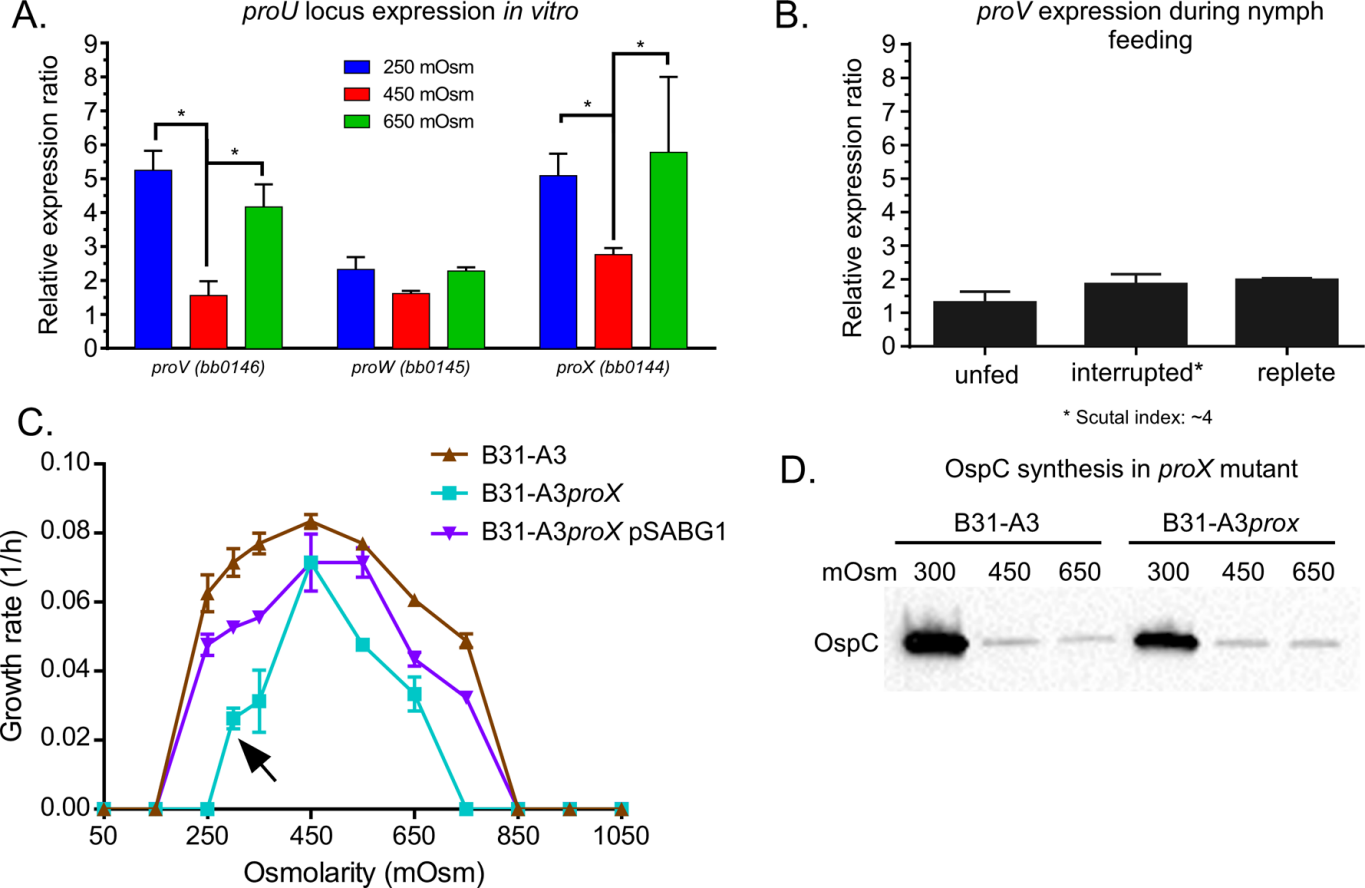
The ProU system and its role in osmotolerance. (A) *In vitro* expression of the three genes of the *proU* locus: *proV*, *proW* and *proX* at 250, 450 and 650 mOsm. Gene expression was normalized to *enoS*. (B) *proV* expression during nymph feeding. (C) Rate of growth of strains B31-A3, B31-A3*proX* and B31-A3*proX* pSABG1 at osmolarities ranging from 150 to 1050 mOsm under microaerobic conditions. The arrow denotes growth at 300 mOsm. (D) Immunoblot of cell lysates (40μg of protein/lane) of *B*. *burgdorferi* B31-A3 and B31-A3*proX* cells grown at 300, 450 and 650 mOsm to mid-log phase and probed with OspC-specific antisera.

Changes in osmolarity affect *B*. *burgdorferi* morphology, motility and virulence factor expression. We next sought to characterize factors demonstrated to aid in osmoadaptation in other bacteria. Osmoadaptation involves both the efflux and influx of osmolytes, as well as ions. Among all of the characterized osmolyte transporters in *E*. *coli* or *B*. *subtilis*, only the ProU system is found in the *B*. *burgdorferi* genome [43]. This system is an ATP-dependent transporter for glycine betaine, proline, and/or choline [44–46]. The ProU locus consists of *proV* (ATP-binding subunit), *proW* (integral membrane protein), and *proX* (periplasmic glycine betaine binding protein) and the genes are found in that order in the *B*. *burgdorferi* genome [43]. In other bacteria, the ProU system protects bacterial cells from high osmolarity by scavenging glycine betaine, proline or choline from the growth media [44–46]. To determine whether the ProU system served as an osmoprotectant system in *B*. *burgdorferi*, we first analyzed the expression of *proV*, *proW*, *proX* at 250, 450 and 650 mOsm (Fig 6A). *proV* and *proX* showed a significant increase in expression at 250 and 650 mOsm when compared to 450 mOsm while *proW* did not change at any osmolarity tested. This may suggest that these genes may be transcribed from different promoters or a full length transcript may be post-transcriptionally modified. We also measured the gene expression of the *proV* gene during nymph feeding (Fig 6B). Surprisingly, *proV* expression remained unchanged at different points of nymph feeding.

To determine if the ProU system played a role in the osmotolerence of *B*. *burgdorferi*, we inactivated the ProU locus by deleting *proX* and tested the *pro* mutant for survival at different osmolarities. We attempted to delete the entire locus (*proX*, *proW* and *proV*) but we were unable to do so, probably because choline is used to synthesize phosphatidylcholine (a major phospholipid in *B*. *burgdorferi* [47]). We grew B31-A3 and B31-A3Δ*proX* at various osmolarities and the *proX* mutant showed a narrower range of osmotolerance than the wild-type (Fig 6C). Because of the effect of the *proX* mutation on growth, we tested the effect of *proX* inactivation on virulence and strain B31-A3*proX* was fully virulent in mice (S1 Table). While the growth rates were slower in this mutant than in the wild-type B31-A3 or the complemented strain B31-A3*proX* pSABG1, B31-A3*proX* cells, at an osmolarity of 300 mOsm (Fig 6C, denoted by the arrow), were motile and showed an increase in the expression of OspC that was characteristic of wild-type cells at lower osmolarity (Fig 6D). Overall, these data suggest that the ProU locus facilitates osmoadaptation but over a very narrow range of osmolarities and glycine betaine, proline or choline do not expand the osmotolerance of *B*. *burgdorferi* cells. It seems remarkable that *B*. *burgdorferi* is so very well adapted to living within a narrow range of osmolarities that directly reflects its immediate environment during the infective cycle.

### *gltP* encoding a putative L-glutamate transporter was regulated by the osmolarity and throughout the enzootic cycle

**Fig 7:**
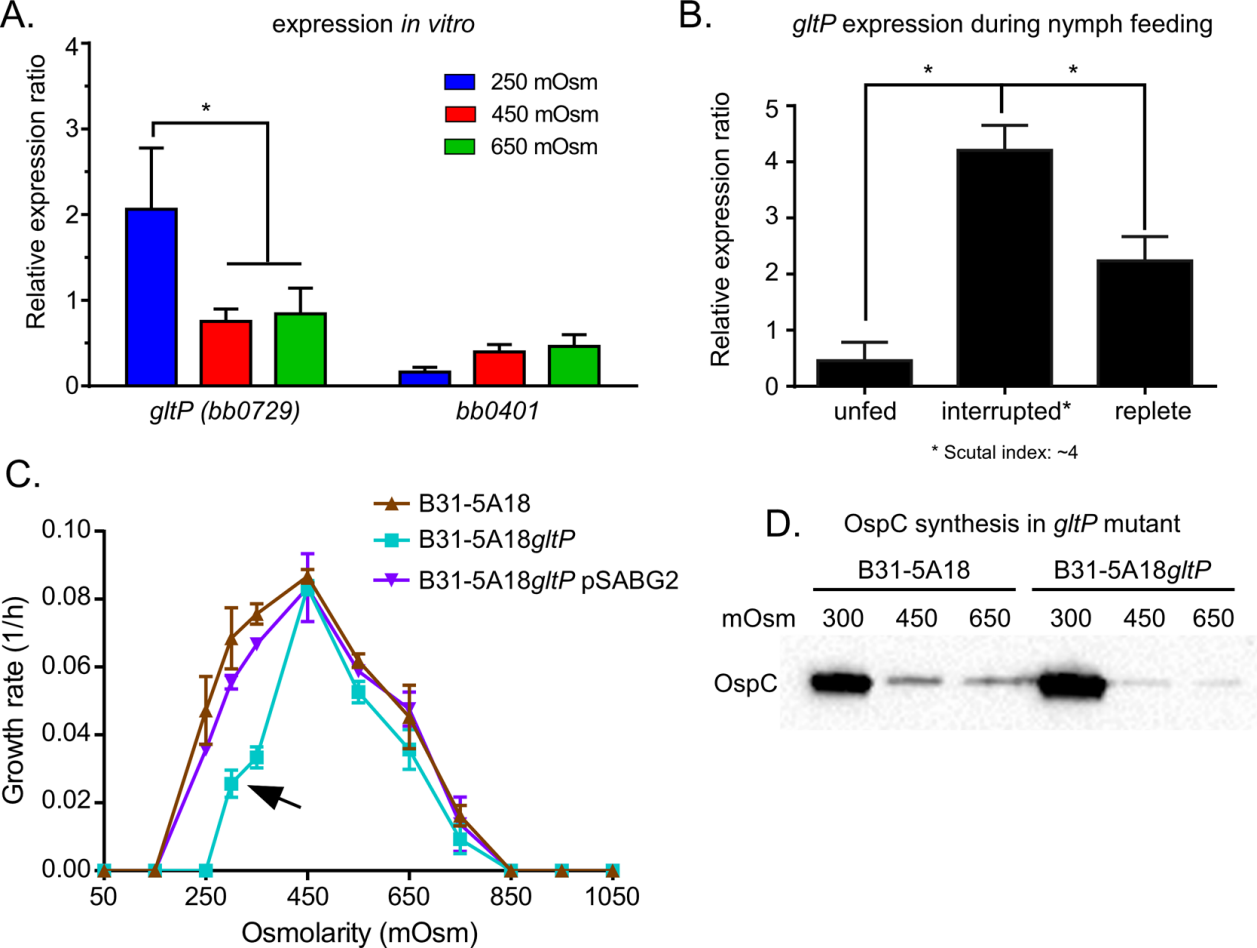
*gltP* and its potential role in osmotolerance. (A) *In vitro* expression of the putative glutamate transporters (*bb0401* and *gltP*). Gene expression was normalized to *enoS*. (B) *gltP* expression during nymph feeding (C). Rate of growth from 150 to 1050 mOsm in microaerobic condition. (D) Immunoblots of *B*. *burgdorferi* B31-5A18 and B31-5A18*gltP* grown at 300, 450 and 650 mOsm to mid-log phase and cell lysates (40μg of protein/lane) probed with OspC-specific antisera.

L-glutamate has been described to be involved in osmoadaptation in bacteria and is readily available in mammalian blood [15, 22, 24, 27, 33, 48]. Classically, bacteria, such as *E*. *coli*, increase the synthesis of L-glutamate to promote growth at high osmolarity (~1000 mOsm) [26, 48, 49]. Because *B*. *burgdoferi* is unable to synthetize L-glutamate [43, 47], we searched for L-glutamate uptake systems in the *B*. *burgdorferi* genome and identified two putative transporters for L-glutamate: *bb0729* (*gltP*) and *bb0401*. Gene expression analyses revealed that only *gltP*, not *bb0401*, was differentially regulated in response to changes in osmolarity, increasing 3-fold at 250 mOsm (Fig 7A). We also measured *gltP* expression before, during and after feeding in nymphs. The expression of *gltP* increased 5.2-fold during feeding at a scutal index of ~4 and decreased 2-fold in replete ticks compared to unfed ticks (Fig 7B). These data suggested that glutamate might function as an osmoprotectant at lower osmolarity.

Because these data suggest a role for L-glutamate as an osmoprotective molecule, we tested this more directly. An insertion inactivation mutant (B31-5A18*gltP*) was obtained from the transposon mutant library [50] and this mutant was tested for growth and survival at different osmolarities. As expected from the expression (Fig 7A), strain B31-5A18*gltP* had a slower growth rate at 300 mOsm (blood osmolarity) than the wild-type strain B31-5A18 or the complemented strain B31-5A18*gltP* pSABG2 (Fig 7C, denoted by the arrow). As was observed in B31-A3*proX*, B31-5A18*gltP* cells, at 300 mOsm, showed normal motility and increased expression of OspC (Fig 7D). The effect of *gltP* inactivation on virulence was tested and, as with B31-A3*proX*, B31-5A18*gltP* was fully virulent in mice (S1 Table). These data showed that: i) *gltP* expression responded to low osmolarity both *in vitro* and *in vivo*, ii) exogenous L-glutamate played a role in osmoprotection at low osmolarity, and iii) L-glutamate transport does not affect survival in mice. Currently, we are trying to test the role of osmoprotectants, such as glutamate, glycine betaine and proline, in ticks by (i) measuring the levels of these molecules in the tick bloodmeal, (ii) generating a B31-A3Δ*gltP*-Δ*proX* double mutant, and (iii) testing all mutants in mice and ticks.

### Low osmolarity altered the expression of ion transport systems *in vitro* and *in vivo*

**Fig 8:**
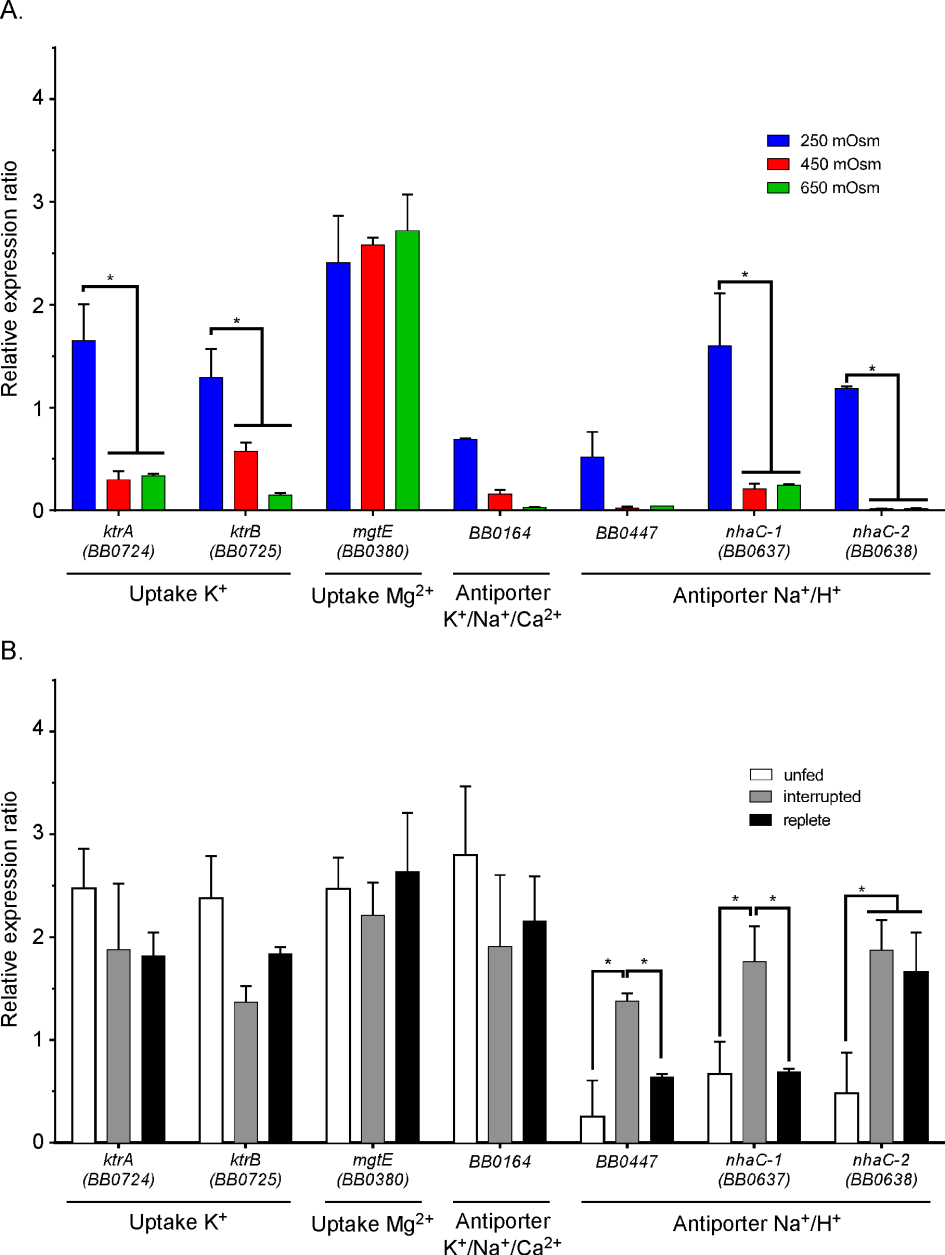
Expression of genes encoding putative ion transporters. (A) Expression analyses of different ion transporter genes of *B*. *burgdorferi* grown in BSK-II at 250, 450 and 650 mOsm. (B) Same as A except measured in unfed, partially fed and replete *I*. *scapularis* infected with *B*. *burgdorferi*. Gene expression was normalized to *enoS*.

To investigate the role of ion transport in osmotolerance, we analyzed the gene expression profiles of ion transport systems identified in the genome of *B*. *burgdorferi* [43](Fig 8A). These included the *ktrAB* transport system (potassium uptake), the K^+^/Na^+^/Ca^2+^ transport system (*bb0164*), the three Na^+^/H^+^ antiporter systems (*bb0447* and *nhaC-1*, *nhaC-2*) and the Mg^2+^ uptake system (*mgtE*, *bb0380*). The expression of both *nhaC-1* and *nhaC-2* increased 10-fold at 250 mOsm osmolarity, suggesting an import of H^+^ and export of Na^+^ was involved in osmoadaptation (Fig 8A). Expression of the K^+^/Na^+^/Ca^2+^ antiporter system increased 3-fold, suggesting an adaptive flux of K^+^, Na^+^ and/or Ca^2+^ (Fig 8A). Furthermore, the expression of the *ktrAB* system increased 4.6-fold suggesting that the flux of K^+^ could augment osmoadaption (Fig 8A). *mgtE* expression was not affected by the changes in osmolarity which was expected since magnesium has never been shown to have a role in osmotolerence (Fig 8A). Taken together, the gene expression data suggest that the flux of ions would promote survival at low osmolarity.

To confirm that the *in vitro* analysis was consistent with observed *in vivo* expression, we analyzed the gene expression of each of the previously mentioned transporters during nymph feeding (Fig 8B). The three Na^+^/H^+^ antiporters (*bb0447*, *nhaC-1*, *nhaC-2*) were induced during the feeding, increasing 8.6-fold, 2.1-fold and 2.7-fold respectively (Fig 8B). The expression of *bb0447* and *nhaC-1* in replete ticks returned to the initial expression level observed in unfed ticks (Fig 8A). Only *nhaC-2* stayed at the levels of expression observed during tick feeding (Fig 8B). Taken together, these data suggest that *B*. *burgdorferi* alters the expression of its ion transport systems which may allow the bacterium to adapt to changing osmotic conditions in the tick midgut during feeding and in its mammalian hosts. It is also possible that other factors such as ion availability (e.g. sodium) may be affecting the regulation of these transport systems.

## Discussion

*B*. *burdorferi* lives in two distinctly different environments: the mammalian host and the tick vector. As the bacteria shuttles back and forth between host and vector, they encounter conditions that are distinct to each setting. For example, when *B*. *burgdorferi* are colonizing a mammalian host, they must switch their surface proteins from OspC to VlsE to evade the host immune system [51, 52]. While the exact signal to trigger this change has not been identified, it is clear that the host immune system provides selective pressure to eliminate bacterial cells that have not made the necessary antigenic changes [52]. Surviving cells colonize immune privileged sites existing in a nutrient rich environment with stable physiological parameters (temperature, pH, oxygen, osmolarity, etc.) Conversely, the tick midgut is the locale where *B*. *burgdorferi* faces a different set of conditions. Physiological conditions change between flattened and feeding ticks but most would hardly be considered to be extreme. For example, temperature (23°–34°C), oxygen (mostly anaerobic to ~2–3% O_2_ during feeding), pH (6.8 in flattened or feeding ticks) do not vary significantly while nutrients (nutrient rich to starvation) and reactive oxygen (ROS) or reactive nitrogen (RNS) species may be considered more variable challenges. What is remarkable is that *B*. *burgdorferi* is very well adapted to these conditions and senses minor changes in the tick “environment” to regulate expression of key virulence factors. In this study, we characterized another physiochemical parameter, osmolarity, that changed during tick feeding and may be a signal triggering the expression of essential virulence factors (e.g., OspC, DbpA, etc.).

As previously described in other species and genera of ticks, osmolarity fluctuates during acquisition of a blood meal [17–20]. This seemed to be the case for *I*. *scapularis*. Midgut contents, isolated from feeding ticks, showed an interesting, triphasic shift from ~600 mOsm to ~300 mOsm returning to ~600 mOsm during the sequential stages of feeding. The physiological reasons for this shift are certainly related to ion and water flux required to balance the effects of non-diffusible or non-transportable anionic polypeptides concentrated in the bloodmeal (Gibbs-Donnan equilibrium) [53]. Clearly, water and ion fluctuations are required for the recycling of water and solutes necessary to generate the amounts of saliva that are required for long-term, successful feeding of *I*. *scapularis*.

Interestingly, experiments on wild-type *B*. *burdorferi* at different osmolarities indicated that the cells had a narrow range of osmotolerance (Fig 2A) compared to *E*. *coli*. Normal doubling times were observed over a range of ~250 to ~650 mOsm under anaerobic and microaerobic conditions, mimicking the conditions observed in the bloodmeal during feeding. The initial observations of cells by dark-field microscopy at different osmolarities indicated that motility was affected as osmolarity reached 650 mOsm. This was of particular interest because Dunham-Ems *et al*. reported that *B*. *burgdorferi* cells have two phases of motility in the midgut of ticks during feeding [37, 54]. Cells were observed to have normal motility and evenly distributed throughout the bloodmeal or were nonmotile and clumped associating with the interior face of the midgut lining. Our observations of the motility of *B*. *burgdorferi* suggest that increased osmolarity may be partially responsible for altered motility observed in feeding ticks [37].

Other interesting trends occurred in *B*. *burgdorferi* cells at physiologically relevant osmolarities. Immunoblots of protein isolated from cells grown at low osmolarity, indicated that the cells increased the expression of virulence related proteins such as OspC, DbpA and BBA66 (Fig 3A). It has been shown that these proteins are required for the successful transmission and survival of *B*. *burgdorferi* in mammalian hosts [6, 7, 14, 55–60]. Analysis by qRT-PCR of RNA isolated from cells grown at 250 mOsm showed an increase in the transcription of *ospC*, *dbpA* and *bba66* correlating with the increase in expression of these proteins in immunoblots. An increase in the expression of *rpoN* and *rpoS* were observed at low osmolarity. Additionally, immunoblot analysis of B31-A3Δ*rpoN* and B31-A3Δ*rpoS* indicated that OspC, DbpA and BBA66 were not induced in these mutants at low osmolarity. Since it has been shown that OspC, DbpA and other virulence factors are controlled by the Rrp2-RpoN-RpoS regulatory cascade, it seems very likely that low osmolarity is directly affecting this regulatory network. It is interesting to note that the increased expression of important virulence factors at low osmolarity corresponds to the osmolarity measured at the midpoint of feeding (Fig 1B). It has been shown that transmission of *B*. *burgdorferi* occurs ~2 days after the initiation of the feeding, which correlates with the drop in osmolarity measured in the bloodmeal of *B*. *burgdorferi* infected ticks. Additionally, these changes were observed in actively growing (mid-log phase), motile cells. It is interesting to speculate that a drop in osmolarity could also serve as a signal to trigger the migration of *B*. *burgdorferi* from the midgut to the hemolymph and ultimately to the salivary glands during feeding. However, at this time, we do not have any direct experimental evidence supporting this hypothesis.

High osmolarity (650 mOsm) occurs in the midgut of an unfed tick, at the initiation of feeding and after feeding is complete. Except for a slight increase in the expression of OspA, the expression of other virulence factors remained unchanged at high osmolarity (650 mOsm) compared to cells grown in BSK-II (450 mOsm) (Fig 3). However, high osmolarity not only affected motility but also had another very interesting effect on *B*. *burgdorferi*. Analysis of a B31-5A4Δ*rrp1* mutant indicated that this strain was exquisitely sensitive to osmolarities >500 mOsm compared to strain B31-5A4 and cells rapidly lysed after less than 4h of exposure to increased osmolarity. Rrp1 is the response regulator in the Hk1-Rrp1 TCS and functions as a di-guanylate cyclase [2–4, 61]. C-di-GMP acts as a secondary messenger for signal transduction in bacteria and the levels of c-di-GMP increased dramatically at high osmolarity (Fig 5D). Rrp1 has also been shown to be required for tick colonization, motility and the regulation of genes involved in glycerol metabolism [2, 3, 62]. In addition to its regulatory functions, Caimano *et al*. showed that B31-5A4Δ*rrp1* was virulent in mice but this mutant rapidly lysed after being acquired by ticks fed on mice infected with this strain [2]. Also, B31-5A4Δ*rrp1* rapidly lysed when introduced into ticks by artificial feeding. Collectively, these data suggest that at high osmolarity, Rrp1: i) was required for survival; ii) had increased diguanylate cyclase activity; iii) is required for tick colonization; and iv) could putatively regulate *B*. *burgdorferi* motility.

Lastly, we investigated the osmoadaptation of *B*. *burgdorferi*. In bacteria, the response to changes in external osmolarity happens at two levels. To restore a conductive intracellular environment, cells transport ionic solutes like K^+^, Na^+^ and compatible solutes glutamate, proline and glycine betaine [22, 23]. At low osmolarity, ionic solutes (primarily K^+^) accumulate while at high osmolarity, compatible solutes accrue to support a high intracellular osmotic pressure without the deleterious effects that ionic solutes have on the activity of metabolic and biosynthetic enzymes [24]. When compatible solutes are not available in the extracellular milieu, the cells will increase their intracellular concentrations by accelerating the synthesis of these important osmoprotectants. Together, these osmoadaptive systems allow bacteria like *E*. *coli* and *Salmonella typhimurium* to tolerate osmolarities from 50–1400 mOsm.

Unlike *E*. *coli* or other spirochetes like *Treponema denticola* and *L*. *interrogans* [22–24, 36], the *B*. *burgdorferi* genome does not harbor the genes encoding proteins to synthesize osmolytes (e.g., proline, choline or glutamate). However, the genome does have three putative osmolyte transport systems: the *proU* system for the transport of glycine betaine, proline or choline, as well as *bb0729* (*gltP*) and *bb0401* both of which are annotated as glutamate transporters. Transcription of the *proU* system increased at low and high osmolarity *in vitro* suggesting that this transport system might be involved in osmoprotection. Additionally, a B31-A3*proX* mutant strain showed a narrower range of osmotolerance than wild-type B31–A3. However, choline, proline and glycine betaine did not increase the range of osmotolerance of B31-A3. These data indicate that these compatible solutes are required for the survival of *B*. *burgdorferi* within the narrow range of osmolarities encountered in the bloodmeal of feeding ticks.

The results for glutamate are distinctly different from what was expected based on previously published information on the role of glutamate in protecting *E*. *coli* and *S*. *typhimurium* at high osmolarity [48]. As previously mentioned, compatible solutes (e.g., glutamate, proline) protect cells at high osmolarity while ionic solutes (e.g., K^+^) protect cells at low osmolarity [22, 27, 32, 34]. The mutant strain B31-5A18*gltP* was more sensitive to low osmolarity while high osmolarity had no effect on the growth and survival of this mutant compared to B31-5A18. Predictably, the expression of the genes encoding ionic solute transport systems such as *ktrAB* (K^+^ transport), *bb0164* (K^+^/Na^+^/Ca^2+^), *bb0447*, *nhaC-1* and *nhaC-2* increased at low osmolarity (250 mOsm). Currently we do not understand why a compatible solute like glutamate is required as an osmoprotectant for *B*. *burgdorferi* at low osmolarity but we suspect that it plays a role in the accumulation of ionic solutes in cells as they respond and adapt to low osmolarity. This may be an important function since it has been shown that >70% of the K^+^ is cycled into the hemolymph and saliva during the feeding of *I*. *ricinus* and *D*. *andersonii* [17–21]. Clearly, the inability of *B*. *burgdorferi* to synthesize compatible solutes has narrowed the limits of their osmotolerance but, despite this, they are finely adapted to the narrow range of osmolarities that they encounter in the tick bloodmeal/midgut and the mammalian host.

While *B*. *burgdorferi* cells are well adapted to a narrow range of osmolarity, what was remarkable was that they were using these relatively small changes in osmolarity as a signal to affect at least two regulatory pathways. First, high osmolarity (650 mOsm) has a dramatic effect on motility in wild-type B31-A3, B31-5A18 and B31-5A4. In addition, strains B31-5A4Δ*hk1* and B31-5A4Δ*rrp1* did not survive at osmolarities above 500 mOsm. As important, the levels of the secondary messenger molecule, c-di-GMP, increased dramatically at high osmolarity, most likely due to an increase in the diguanylate cyclase activity of Rrp1 [63]. These data suggest a role for Hk1 and Rrp1 in the adaptation to and survival of *B*. *burgdorferi* cells at osmolarities of 600 to 650 mOsm. Second, analyses of protein and gene expression in B31-A3, B31-A3Δ*rpoN* and B31–A3Δ*rpoS* suggested that *B*. *burgdorferi* cells express key virulence factors, such as OspC, DbpA and BBA66 at low osmolarity and this increase in expression was dependent on RpoN and RpoS.

**Fig 9:**
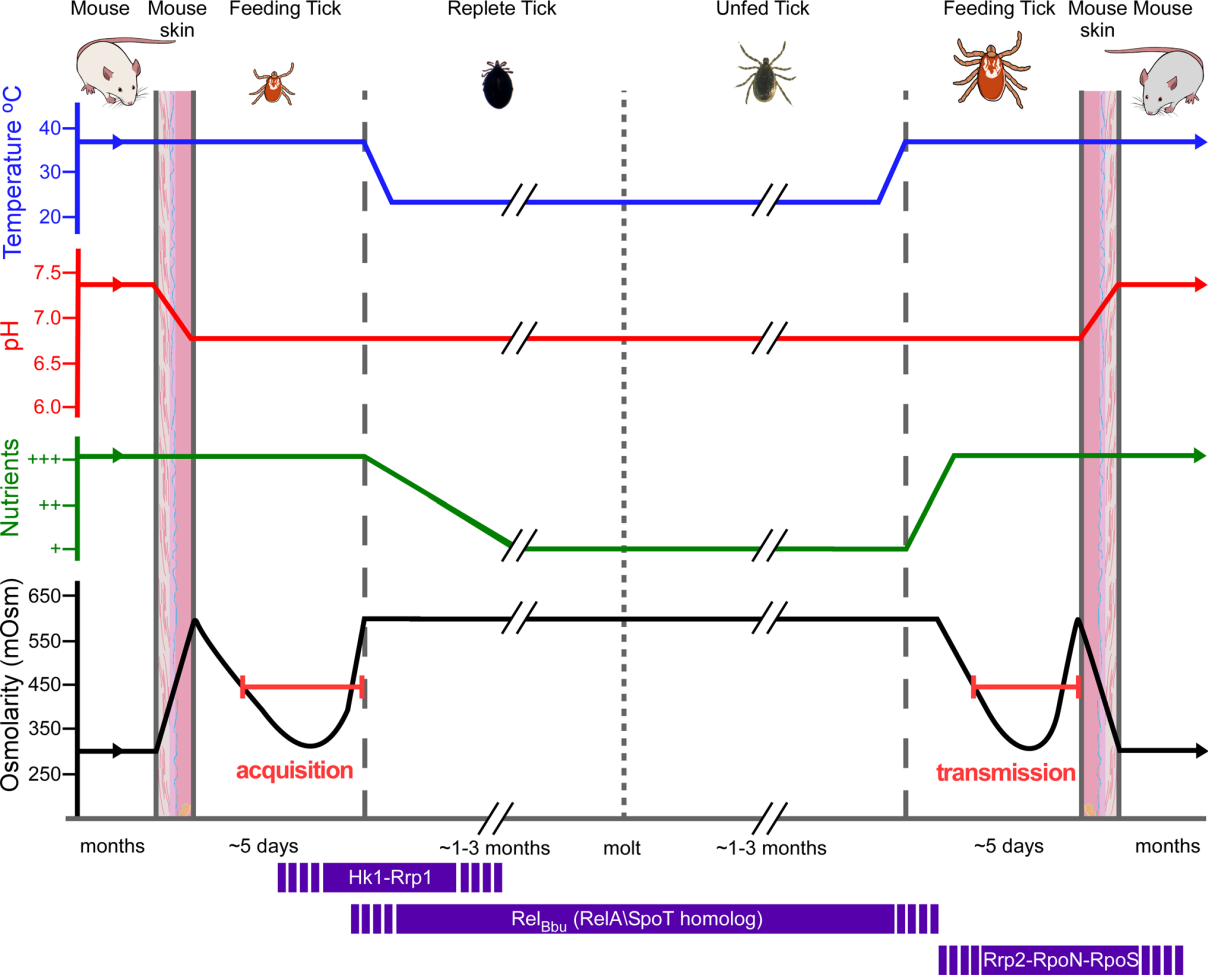
Model for generation of osmotic stress and its effect on regulatory pathway during the enzootic cycle. During acquisition, spirochetes encounter an increase of the osmolarity, which requires gene regulation by Hk1-Rrp1. The activation of Hk1-Rrp1 system increases the level of c-di-GMP which triggers metabolic adaptation to the new environment and also affects cell motility [7, 37, 62]. After feeding, the depletion of nutrients activates Rel_Bbu_-dependent gene expression which promotes long-term survival in the midgut [3]. During the second feeding, Rel_Bbu_ regulatory effects decrease, restoring normal growth in a nutrient replenished environment. Other relevant physiological changes (decreasing osmolarity, increased temperature, etc.) stimulate Rrp2-RpoN-RpoS-dependent virulence factors required for the transmission and successful colonization of a new host.

Our current working model (Fig 9) is that as *B*. *burgdorferi* cells are acquired by feeding ticks, they rapidly transition from osmolarities of ~300 mOsm in mammalian blood and tissue to ~600 mOsm in the tick midgut at the beginning of feeding. It seems very likely that Hk1-Rrp1 and c-di-GMP are essential for this transition. At the end of acquisition, in replete ticks, the osmolarity returns to ~600 mOsm and motility is impaired, potentially limiting spread of *B*. *burgdorferi* and trapping them in the midgut. Long-term survival of cells through the molt is most likely mediated by Rel_Bbu_ (RelA/SpoT homolog) [5]. At the midpoint of the second feeding, as the osmolarity cycles from ~600 to ~250, motility increases and the cells respond to lower osmolarity by expressing ionic solute transport systems. Most importantly, the RpoN-RpoS regulatory cascade is also stimulated by low osmolarity and triggers the expression of vertebrate virulence-related proteins. At this point, the cells are actively growing, have normal motility and are expressing proteins necessary to promote successful transmission to the next mammalian host. It seems clear that changing osmolarity can affect two different regulatory pathways, Hk1-Rrp1 and RpoN-RpoS, and is potentially a major signal sensed by *B*. *burgdorferi* during acquisition and transmission.

## Materials and Methods

### Bacterial strains, media and growth conditions

The strains used in this study are described in the S2 Table. *B*. *burgdorferi* strains were grown in BSK-II medium, pH 6.8 at 34°C [64] under microaerobic environment (5% O_2_, 5% CO_2_) and, when indicated, under anaerobic (5% CO_2_, 5% H_2_, balance N_2_) or aerobic condition. Cell densities were determined by dark-field microscopy (Eclipse E600, Nikon, Melville, NY). The osmolarity of the BSK-II medium is 450 mOsm. To obtain high-osmolarity medium, NaCl was added. To obtain low-osmolarity medium, ddH_2_O was added. BSK-II medium for plating contained 0.6% agarose. Importantly, low-osmolarity BSK-II media was tested to ensure that essential nutrients were not too dilute to support normal growth. This was accomplished by adding NaCl to the dilute BSK-II to adjust the osmolarity to 450 mOsm. Restoring the osmolarity of diluted BSK-II to 450 mOsm restored normal growth of wild-type B31-A3 [65]. For survival assays, various wild-type and mutant strains were grown in BSK-II medium at different osmolarities starting at 1 x10^5^ cells/ml to early stationary phase of growth. Every 24 h an aliquot of each culture was examined by dark-field microscopy and plated on BSK-II. Plates were incubated at 34°C under microaerobic conditions for 7–14 days to allow enumeration of CFU. The cell length (40 cells per slide, 5 slides from 5 independent cultures) was measured using ImageJ software.

*E*. *coli* strains were grown in Lysogeny broth [66] or in low osmolarity medium, called LOS (4 g of casein hydrolysate, 0.5 mg of FeSO_4_, 18 mg of MgCl_2_, 200 mg of (NH_4_)_2_SO_4_ and 175 mg of K_2_HPO_4_ per liter, pH 7.2) [67]. The LOS medium osmolarity is 70 mOsm. To obtain high-osmolarity medium, NaCl was added.

Growth rates were defined during the exponential phase [68]. Briefly, the growth rate is defined by 1/doubling time and expressed in 1/h.

### Measure of the osmolarity

All osmolarities were measured with a Wescor vapor pressure osmometer at 21°C (model 5500, Wescor, Inc., Logan UT, USA) and expressed as milli-osmolar (mOsm).

### Construction of the *proX*::*himar1*-Gm mutations and complemented strains

The proX::*himar1*-Gm was amplified by PCR from B31-5A18 NP1 proX::*himar1*-Gm (proUF ACAGATGAGGTTGTAGCAGCA and proUR GCATATACAAACCTACCTGCTC) and cloned into TopoZeroBlunt (Invitrogen, Carlsbad, CA) to obtain Topo0ProX::Gm vector. The resulting plasmid was transformed into low-passage *B*. *burgdorferi* B31-A3 strain as described previously [69] and gentamicin-resistant colonies were analyzed by PCR to confirm the inactivation of *proX*. Mutants were screened using plasmid specific primer sets [25]. Mutant strain B31-A3*proX* harbored all plasmids except cp9 was used for further characterization.

For the complementation, the *proU* operon was amplified by PCR using proUF and proUR primers and cloned into PCR-XL-TOPO following the manufacturer’s recommendations (Invitrogen, Carlsbad, CA). The resulting plasmid was digested with SacI-PstI and the *proU* fragment was cloned into the pKFSS1 [70] shuttle vector digested with the same restriction enzyme to obtain pSABG1.

The *gltP* gene was synthesized by Genscript, USA and cloned into the pKFSS1 shuttle vector digested with SacI-PstI to obtain pSABG2. The resulting plasmids were transformed into low-passage *B*. *burgdorferi* mutants strains as described previously [69] and spectinomycin-resistant colonies were analyzed by PCR to confirm the construction.

### RNA extraction, reverse-transcription and Q-PCR

RNA samples were extracted from *B*. *burgdorferi* cultures using the RNeasy mini kit (Qiagen, Valencia, CA) according to the manufacturer’s protocol. Three independent cultures were used for each osmolarity. Total RNA from ticks was isolated from 3 pools of 7 nymphs fed on mice infected by needle inoculation with B31-A3. RNA samples was extracted using RNeasy mini kit (Qiagen, Valencia, CA). Ticks were frozen at -80°C directly and crushed. TRIzol (Life technologies, Carlsbad, CA) was added with chloroform. After centrifugation, the upper phase was mixed with ethanol 70% (1:1) and loaded onto the provided Qiagen column according to the manufacturer’s instructions. Digestion of the genomic DNA was performed using TURBO DNA-free DNase I (Life Technologies, Carlsbad, CA). The cDNA was synthesized using the Superscript III reverse transcriptase with random primers (Invitrogen, Carlsbad, CA). To determine gene expression levels, a relative quantification method was employed using the *enoS* gene as a reference gene (S3 Fig). All samples were performed in at least three biological replicates and three technical replicates on a Roche LightCycler 480 System using Green PCR Master Mix (Life technologies, Carlsbad, CA). All primers used for the study are listed in S3 Table. To determine relative gene expression, the LightCycler 480 software version 1.5 was used. The relative quantification was performed following the E-Method using the *enoS* as a housekeeping gene [71].

### SDS-PAGE and immunoblots

For analysis of cell lysates by Western-blot, bacteria were grown to mid-log phase at 34°C in microaerobic conditions. The cells were harvested by centrifugation, washed twice in HN buffer (50 mM HEPES pH7.5, 50 mM NaCl), resuspended in 0.25M Tris-HCl pH 6.8 and lysed by sonication. The protein concentration was determined with Take3 micro-volume plate in a Synergy 2 Multi-Mode plate reader (BioTek Instruments, Winooski, VT, USA). 40 μg of protein was loaded in a 4–20% pre-cast SDS-PAGE gel (Bio-Rad, Hercules CA, USA) and transferred to a nitrocellulose membrane using a Trans-Blot TurboTM blotting system (Bio-Rad, Hercules CA, USA) with a pre-programmed protocol (2.5A, up to 25V, 3 min). Western blotting was performed using standard protocols, *i*.*e*. membrane blocking 1 h in 5% nonfat milk in PBS-T (0.1% Tween 20), then incubating 1hr in PBS-T with primary antibodies, washing in PBS-T and then incubating 30 min in PBS-T with Rec Protein A-HRP (1:4,000; Life technologies, Carlsbad, CA, USA) or with the anti-IgY conjugated to HRP (1:50,000; for α-BBA66, Aves Laboratories, Tigard, OR, USA). For the primary antibodies, the following dilutions were used: α-OspC 1:1,000 [72], α-DbpA purified antibody 1:1,000 (Rockland Immunochemicals, Gilbertsville, PA, USA), α-BBA66 1:4,000 [73], α-RpoS 1:500 [74], α-RpoN 1:1,000, α-BosR 1:500, α-OspA 1:2000 (Rockland Immunochemicals, Gilbertsville, PA, USA), α-Rrp1 1:1,000, α-Rrp2 1:2,000 or infected-mouse serum 1:200 (mice infected with wild-type B31-A3 spirochetes by tick bite). Blots were imaged by chemiluminescent detection using Super Signal Pico chemiluminescent substrate kit (Thermo Scientific, Rockford, IL, USA).

Rabbit polyclonal antisera directed against Rrp2 or BosR protein was prepared according to a previously published protocol [72]. Rabbit polyclonal antisera directed against Rrp1 protein was prepared by Rockland Immunochemicals, Gilbertsville, PA, USA.

### Tick feeding

*I*. *scapularis* egg masses (Oklahoma State University) were allowed to hatch and mature in a controlled temperature, humidity and photoperiod environment. RML mice were needle inoculated by intradermal injection with 100 μl of BSK-II containing 1 x 10^5^ *B*. *burgdorferi* B31-A3 and after three weeks, infection confirmed by culturing ear punch biopsies. Larval ticks were fed to repletion on infected mice (naïve mice for non-infected cohort), collected and allowed to molt into nymphs and cure in a controlled environment. Nymphal ticks were then fed on naïve RML mice and mechanically removed periodically during the feeding and further processed for osmolarity measurement or RNA isolation as indicated. For infected nymphs, mice were sacrificed 3–6 weeks post inoculation and tissues (ankle joint, bladder and ear) were cultured to verify infection of the ticks through transmission to the naïve animal. Several of the nymphs (infected and non-infected cohorts) were fed to repletion, collected and allowed to molt into adults. After maturation, these ticks were fed on New Zealand White rabbits. Ticks were removed during feeding and further processed for osmolarity determination or RNA extraction as indicated.

### Measure of c-di-GMP

c-di-GMP was quantified from *B*. *burgdorferi* cultures using the cGMP Direct Biotrak EIA (GE Healthcare, UK) according to the manufacturer’s protocol. Four independent culture samples were used for each condition. Protein was quantified using a Microplate with Synergy 2 plate reader (BioTek, VT, USA)

### Scutal index measurement

Scutal index in feeding ticks was determined as previously described [35]. Briefly, for nymphs, the width of the scutum and length of the body (Fig 1A) were measured under a dissecting microscope configured with an ocular micrometer calibrated to a stage micrometer at a given magnification. For adult ticks, a similar procedure was performed except that a hand held magnifying micrometer was used. Because the width of the scutum remains constant and the length of the body increases proportionately during tick feeding, its ratio provides the most reliable and reproducible indicator of feeding progress.

### Mouse infection

In triplicate, RML mice were inoculated intradermally with 1 x 10^5^ cells in 100 μl BSK-ll with *B*. *burgdorferi* strains B31-A3, B31-5A18, B31-A3*proX* and B31-5A18*gltP*. Four weeks post-infection, the mice were sacrificed, tissues dissected (ankle joint, bladder and ear) and cultured in BSK-II to confirm the presence of spirochetes. Rocky Mountain Laboratories (RML), NIAID, NIH in Hamilton, MT are accredited by the International Association for Assessment and Accreditation of Laboratory Animal Care.

### Recovery of tick midgut contents

The blood meal from the midgut of fed *Ixodes scapularis* adults and nymphs was collected from interrupted and replete ticks using the following methods. Ticks were held behind the basis capituli with fine pointed forceps. With a second set of forceps, the abdomen was pierced and the contents extruded with slight downward pressure into a microfuge tube. Adults were collected individually and nymphs of similar scutal index were pooled to provide sufficient sample subsequent for analyses.

### Ethics statement

Mouse infection studies were carried out in accordance with the Animal Welfare Act (AWA 1990), the guidelines of the National Institutes of Health, Public Health Service Policy on Humane Care (PHS 2002) and Use of Laboratory Animals and the United States Institute of Laboratory Animal Resources, National Research Council, Guide for the Care and Use of Laboratory Animals. All animal work was done according to protocols approved by the Rocky Mountain Laboratories, NIAID, NIH Animal Care and Use Committee (Protocol Number 2014–021). The Rocky Mountain Laboratories are accredited by the International Association for Assessment and Accreditation of Laboratory Animal Care (AAALAC). All efforts were made to minimize animal suffering.

### Statistical analysis

Prism 6 software (v6.00, GraphPad, San Diego, CA) was used for all statistical analyses. The data were analyzed using an unpaired *t* test. P<0.05 was considered significant.

## Supporting Information

S1 Fig Growth of B31-A3 in a range of osmolarities. Growth curves of strain B31-A3 in BSK-II at various osmolarities (mOsm) in microaerobic conditions. Cells were quantified by plating in BSK-II plating.
(TIFF)

S2 Fig The effects of osmolarity on specific proteins regulated by the RpoN-RpoS regulatory cascade. *B*. *burgdorferi* strains B31-A3, B31-A3Δ*rpoN* and B31-A3Δ*rpoS* were grown in 250, 450 and 650 mOsm BSK-II to mid-log phase and cell lysates (40 μg of protein/lane) were subjected by SDS-PAGE and coomassie blue staining.
(TIF)

S3 Fig Absolute quantification of *enoS* and *flaB* by qRT-PCR. The expression of *enoS* and *flaB* analyzed by qRT-PCR in *B*. *burgdorferi* B31-A3. RNA isolated from cells grown at 250, 450 and 650 mOsm. See Methods for RNA extraction and qRT-PCR. To determine absolute quantification, 2^nd^ derivative max methods from the LightCycler 480 software version 1.5 was used.
(TIFF)

S1 Table Mice infectivity of the *proU* and *gltP* mutants. (DOCX)

S2 Table Strains and plasmids of *B*. *burgdorferi* and *E*. *coli*. (DOCX)

S3 Table Q-PCR primer. (DOCX)
