# Supplementary figures and images for: Two Different Virulence-Related Regulatory Pathways in Borrelia burgdorferi Are Directly Affected by Osmotic Fluxes in the Blood Meal of Feeding Ixodes Ticks
Source: PLoS Pathog. 2016 Aug 15;12(8):e1005791. doi: 10.1371/journal.ppat.1005791 (PMC4985143; doi:10.1371/journal.ppat.1005791)

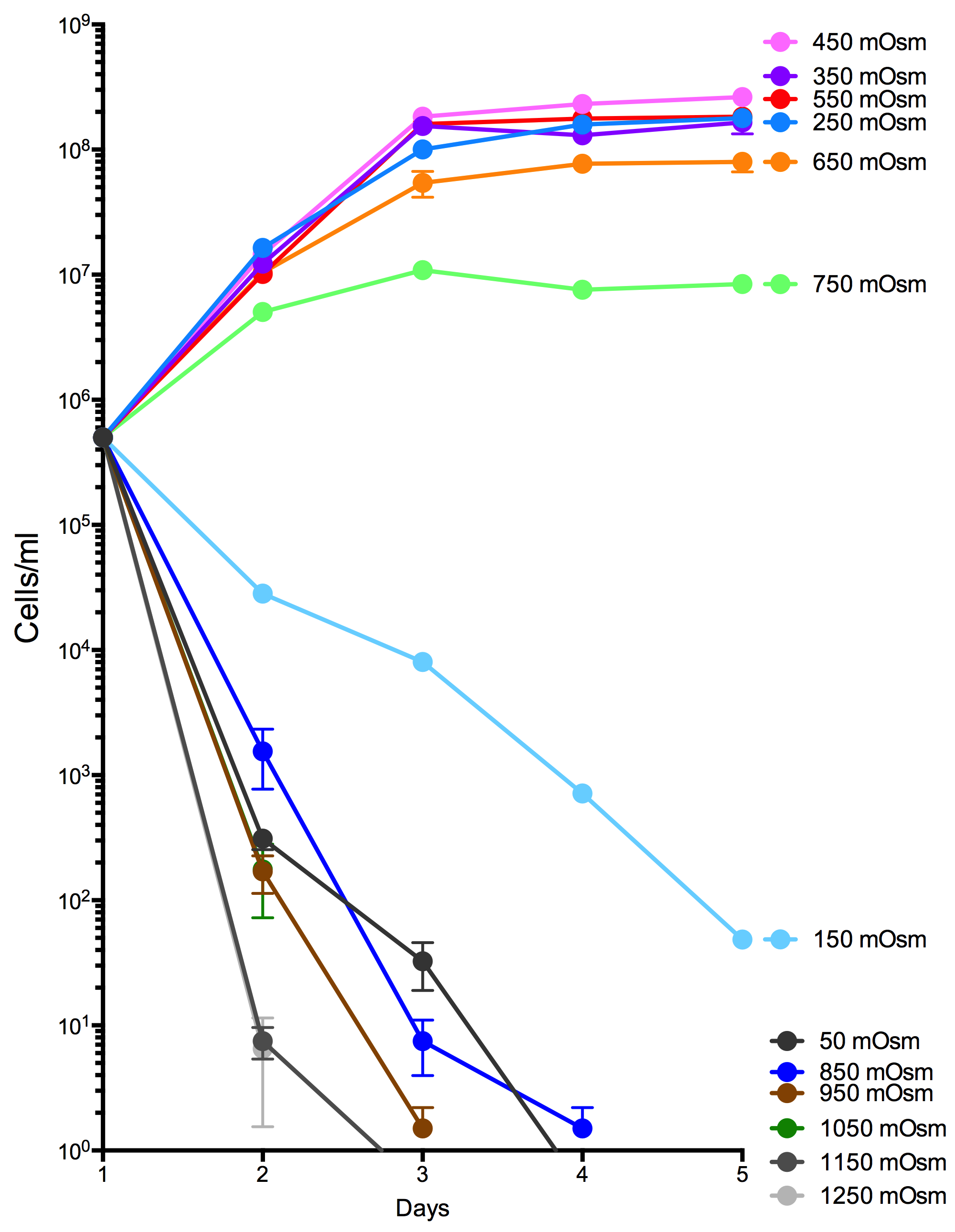

Supplement: S1 Fig — Growth curves of strain B31-A3 in BSK-II at various osmolarities (mOsm) in microaerobic conditions. Cells were quantified by plating in BSK-II plating. (TIFF) [file ppat.1005791.s001.tiff]

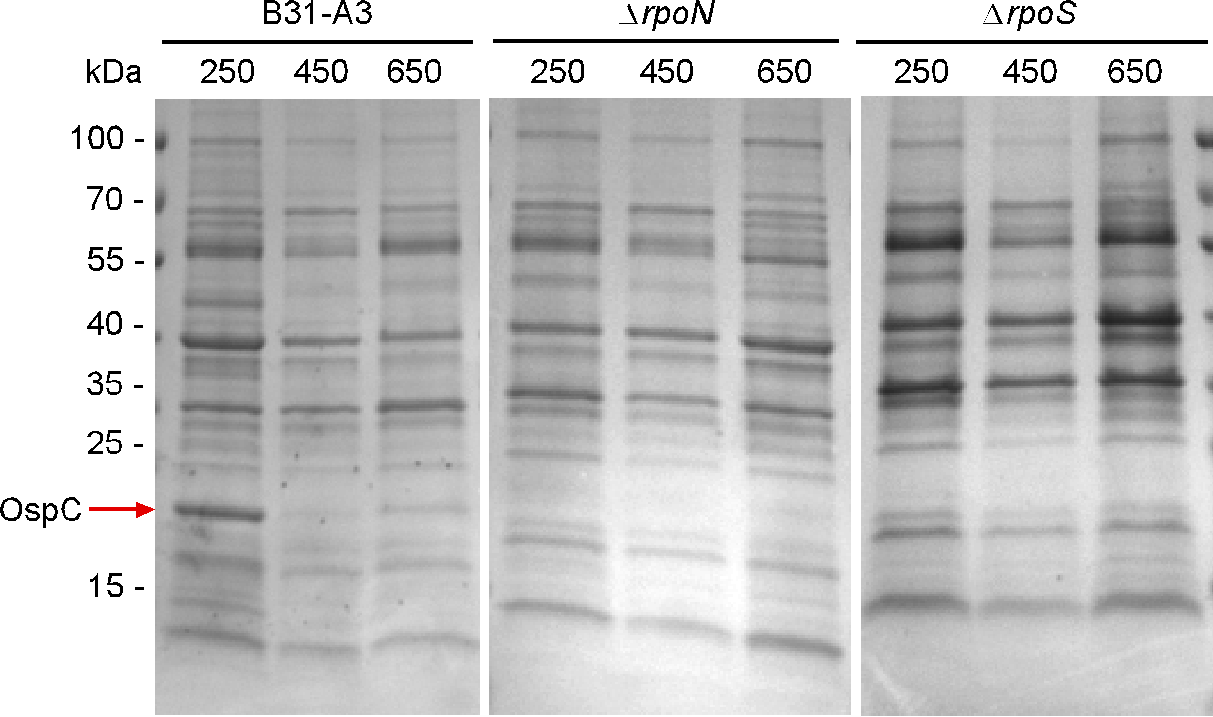

Supplement: S2 Fig — B. burgdorferi strains B31-A3, B31-A3ΔrpoN and B31-A3ΔrpoS were grown in 250, 450 and 650 mOsm BSK-II to mid-log phase and cell lysates (40 μg of protein/lane) were subjected by SDS-PAGE and coomassie blue staining. (TIF) [file ppat.1005791.s002.tif]

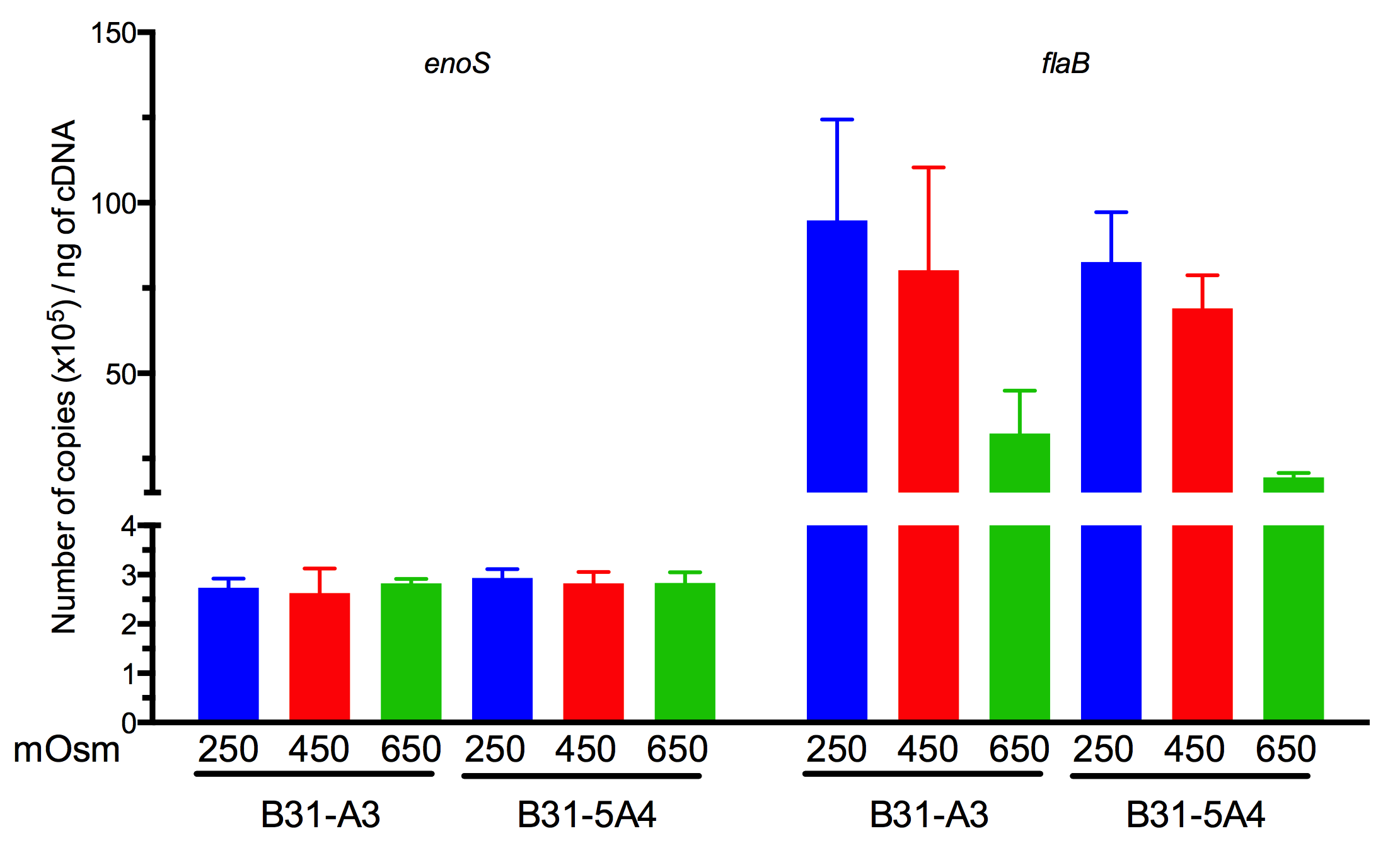

Supplement: S3 Fig — The expression of enoS and flaB analyzed by qRT-PCR in B. burgdorferi B31-A3. RNA isolated from cells grown at 250, 450 and 650 mOsm. See Methods for RNA extraction and qRT-PCR. To determine absolute quantification, 2nd derivative max methods from the LightCycler 480 software version 1.5 was used. (TIFF) [file ppat.1005791.s003.tiff]
