# Supplementary material for: Two Different Virulence-Related Regulatory Pathways in Borrelia burgdorferi Are Directly Affected by Osmotic Fluxes in the Blood Meal of Feeding Ixodes Ticks
Source: PLoS Pathog. 2016 Aug 15;12(8):e1005791. doi: 10.1371/journal.ppat.1005791 (PMC4985143; doi:10.1371/journal.ppat.1005791)
Supplement: S1 Table — (DOCX) [file ppat.1005791.s004.docx]

**Table S1: Mice infectivity of the *proU* and *gltP* mutants**

| Strains | # of mouse tissues culture positive/total | | | # of mice infected/total # of mice |
| --- | --- | --- | --- | --- |
|  | Ear | Joint | Bladder |  |
| B31-A3 | 3/3 | 3/3 | 3/3 | 3/3 |
| B3- A3*proX* | 3/3 | 3/3 | 3/3 | 3/3 |
| B31-5A18 | 3/3 | 3/3 | 3/3 | 3/3 |
| B31-5A18*gltP* | 3/3 | 3/3 | 3/3 | 3/3 |
| B31-5A18*proX* | 3/3 | 3/3 | 3/3 | 3/3 |
