# Supplementary material for: Two Different Virulence-Related Regulatory Pathways in Borrelia burgdorferi Are Directly Affected by Osmotic Fluxes in the Blood Meal of Feeding Ixodes Ticks
Source: PLoS Pathog. 2016 Aug 15;12(8):e1005791. doi: 10.1371/journal.ppat.1005791 (PMC4985143; doi:10.1371/journal.ppat.1005791)
Supplement: S2 Table — (DOCX) [file ppat.1005791.s005.docx]

**Table S2: Strains and plasmids of *B. burgdorferi* and *E. coli***

| Strain/genotype^a^ | Source or reference |
| --- | --- |
| *B. burgdorferi* |  |
| B31-A3 | [64] |
| B31-A3*proX*::*himar1*-Gm | This study |
| B31-5A18 | [50] |
| B31-5A18*gltP*::*himar1*-Gm | [50] |
| B31-5A18*proX*::*himar1*-Gm | [50] |
| B31-5A4  B31-5A4Δ*hk1*  B31-5A4Δ*rrp1*  B31-A3Δ*rpoS*  B31-A3Δ*rpoN*  *E. coli* | [2]  [2]  [62]  [39]  [74] |
| Top10: *mcrA,* Δ*(mrr-hsdRMS-mcrBC),* Φ80*lacZΔ*M15, Δ*lacX74, recA1, araD139,* Δ*(ara- leu)7697, galU, galK, rpsL, endA1, nupG* | Invitrogen |
|  |  |
| Plasmids | |
| pSABG1: pKFSS1-proX | This study |
| pSABG2: pKFSS1-gltP | This study |
| pKFSS1 | [70] |

^a^*Himar1* is a transposon. Gm: gentamicin
