# Supplementary material for: Two Different Virulence-Related Regulatory Pathways in Borrelia burgdorferi Are Directly Affected by Osmotic Fluxes in the Blood Meal of Feeding Ixodes Ticks
Source: PLoS Pathog. 2016 Aug 15;12(8):e1005791. doi: 10.1371/journal.ppat.1005791 (PMC4985143; doi:10.1371/journal.ppat.1005791)
Supplement: S3 Table — (DOCX) [file ppat.1005791.s006.docx]

**Table S3: Q-PCR primer**

| Primer | Sequence | Efficiency |
| --- | --- | --- |
| Qhk1F [2] | CGTCAATTTATTTTCTAAGGATATTTTC | 1.503 |
| Qhk1R [2] | TGCTTCGTCTTCAATTTCACT |  |
| Qrrp1F [2] | AAGGTGCTTACGAGATTGAG | 1.792 |
| Qrrp1R [2] | TCTGTGGAACTTCTTGAACTAA |  |
| QbosRF | TCCACCCTATTCAACTTGACGATATTA | 1.873 |
| QbosRR | CCCTGAGTAAATGATTTCAATAGATTTTG |  |
| Qrrp2F | TTGCGCAGCACTTTCTGA | 1.858 |
| Qrrp2R | TGTGCCTTTGTTTGCAAGTTC |  |
| QrpoNF | CGTTTGGGTCTTTAAATTCG | 1.837 |
| QrpoNR | AACACGGCTTTAGAAATTATCA |  |
| QrpoSF | CTGGACAAAGAAATAGAGGGATCTG | 1.838 |
| QrpoSR | CAAGGGTAATTTCAGGGTTAAAAGAA |  |
| QospCF | TGGTACTAAAACTAAAGGTGCTGAAGAA | 1.951 |
| QospCR | GCATCTCTTTAGCTGCTTTTGACA |  |
| QdbpAF | AGAAACTGGAAGTAGTGGTGAAT | 1.761 |
| QdbpAR | GCACTCCTTGAGCTGTAGTT |  |
| Qbba66F | CTGCATTTGAACTGCTAGATGTTAT | 1.903 |
| Qbba66R | TTCTGCTTCTGGTGTGTTAGAG |  |
| Qbb844F | TGGACTTAGCGAGAGAGCAT | 1.929 |
| Qbb844R | GCCTGTATTGCTAAATTACTATGTGTTC |  |
| QospAF | CTGACAGTAGTGCTGCTACTAAA | 1.675 |
| QospAR | GGTGCCATTTGAGTCGTATTG |  |
| QrpoDF | CTGATCAAGCTCGCACAATTAG | 1.694 |
| QrpoDR | TCTGTGGGATCTTTGCCTAAA |  |
| QproVF | GTGCCAAGCTATGTTCCAATTTC | 1.732 |
| QproVR | CACCAGCATCTATGCCAATCA |  |
| QproWF | TTTGGAATGGGTACAGCTTCT | 1.819 |
| QproWR | CAAAGGACTTAGCTGCCTCTAT |  |
| QproXF | CTGGAGGAATGAAACAAAGAGT | 1.888 |
| QproXR | ATTAAAGGATCAAGTGCCGAAA |  |
| QgltPF | TCGAGTGCTGCAACCATAC | 1.695 |
| QgltPR | GCCCAATTGATGTTCCAAAGG |  |
| Qbb401F | AAAGGCAGAATAGCTAGAGAAC | 1.738 |
| Qbb401R | TGCAGCGTAATTTGCTGTTA |  |
| QktrAF | GCTGAAACTGTTATTCCCAAAGA | 1.586 |
| QktrAR | TCGCCATCAACAAATTCATACC |  |
| QktrBF | GATCTGGCAATTGGAAGGTTATTG | 1.813 |
| QktrBR | CCCTATTCGTCCTGCAAACA |  |
| QmgtEF | GCTCTTGGTACTGTCAAGGTAAA | 1.824 |
| QmgtER | AGCTTATCAGAATGGTGTGGAG |  |
| Qbb164F | GAGCTCTTTACATTGCCAACAA | 1.974 |
| Qbb164R | GCCAAATGCAATGTCTGATTCT |  |
| Qbb447F | GTGTCACCTTAGCAACCGTAATA | 1.829 |
| Qbb447R | GACATAGCAAGTCCAAATACATAAGC |  |
| QnhaC-1F | GTGTGGAGCTATGTTTGGAGATA | 1.768 |
| QnhaC-1R | AAGTTAGTATGGCGGATGGAAA |  |
| QnhaC-2F | CGGAATTTGTCATTGTTGTCTTAGT | 1.745 |
| QnhaC-2R | AGCCGCAATACGCTTTGA |  |
| QflaBF | TGATTAGCCTGCGCAATCATT | 1.796 |
| QflaBR | AATGACAGATGAGGTTGTAGCAGC |  |
| QenoSF | GTGCACACTCTGACAACTCT | 1.927 |
| QenoSR | ACCTCTGCTGCCATTCTTATT |  |
